# Psychological distress and mental health trajectories during the COVID-19 pandemic in Argentina: a longitudinal study

**DOI:** 10.1038/s41598-022-09663-2

**Published:** 2022-04-04

**Authors:** Rodrigo S. Fernández, Lucia Crivelli, Nahuel Magrath Guimet, Ricardo F. Allegri, Soledad Picco, Maria E. Pedreira

**Affiliations:** 1 Instituto de Fisiología, Biología Molecular y Neurociencias (IFIBYNE-CONICET), Buenos Aires, Argentina; 2 Facultad de Ciencias Exactas y Naturales, Universidad de Buenos Aires, Buenos Aires, Argentina; 3 Department of Cognitive Neurology, Neuropsychiatry and Neuropsychology, Fleni, Buenos Aires, Argentina; 4 Global Brain Health Institute (GBHI), San Francisco, USA; 5 Laboratorio de Neurociencia de la Memoria, Facultad de Ciencias Exactas y Naturales, Universidad de Buenos Aires IFIByNE, CONICET, Ciudad Universitaria, C1428EHA Buenos Aires, Argentina

**Keywords:** Psychiatric disorders, Psychology

## Abstract

Psychological-distress increased at the onset of the COVID-19 pandemic in Argentina. Longitudinal studies in developing countries are scarce. Particularly, Argentina had one of the longest lockdowns. Differences in preventive measures against the virus spread between countries may differentially affect the mental health of the populations. Here we aimed to characterize distinct psychological-distress and related-symptoms trajectories associated with the pandemic and explore risk/protective factors. In this longitudinal study, data from 832 Argentineans were collected every 3–5 months, between April 2020–August 2021. Mean psychological-distress levels and related-symptoms tended to increase over time. However, latent-class analysis identified four distinct psychological-distress trajectories. Most individuals had consistently good mental health (Resilient). Two classes showed psychological-distress worsening during the initial phase of the pandemic and recovered at different time points (Fast Recovery; Slow Recovery). The remaining class maintained a mild -level of psychological-distress and began to deteriorate in March 2021 (Deteriorating) continuously. Individuals who are younger, female, have pre-existing psychiatric diagnoses, or have high neuroticism or lower resilience were more likely to experiencing fluctuations in psychological-distress. The mental health trajectory during the pandemic had a complex dynamic. Although most participants remained resilient, a vulnerable group was detected, which deteriorated over time and should be considered by health-services.

## Introduction

In early 2020, the appearance of the novel Coronavirus Disease 2019 (COVID-19) rapidly spread and caused severe health and economic consequences around the globe^1–3^. The Covid-19 pandemic and lockdown measures represented a severe and sustained stressor for societies, which raised severe concerns regarding its effects on general well-being and mental health^4,5^. Several cross-sectional studies around the world showed that social isolation and lockdown measures at the initial phase of the pandemic worsened mental health and increased psychological distress, such as higher rates of anxiety, stress-related symptoms, and depressive symptoms^6–11^. A recent report estimated a global increase in the prevalence of major depressive disorder of 27.6% and 25.6% for anxiety disorders^12^. Common risk factors for poorer mental health during the early stages of the pandemic were proposed, such as being a woman, being young, having a previous mental health diagnosis, lower education, and a lower socioeconomic status^10,13–17^. Moreover, trait-measures such as neuroticism, copying-style and resilience, and state-measures such as COVID-19 related fear were found to modulate mental health outcomes^13,18–20^.

As psychological distress may represent an adaptation to environmental threats^21^, it is unknown whether mental health worsening during the initial phase of the COVID-19 pandemic could be transitory or chronic. There is evidence that sustained stressors such as natural disasters may have long-lasting consequences on mental health^22,23^. However, other studies showed that most people were resilient in the long-term, or their mental health improved following an initial deterioration^24,25^. These previous findings are consistent with the idea that stress promotes adaptation and that most individuals are able to cope with negative or threatening experiences. Longitudinal studies performed in developed countries (i.e. Germany, USA, or United Kingdom) within the first six months of the pandemic, reported that the highest levels of anxiety, depression and psychological distress were observed during the early phases of the pandemic and lockdowns^26–29^. Notably, psychological distress levels tended to decrease or return to pre-pandemic levels as the pandemic unfolded or the restrictive measures were relaxed^26–28,30–33^ [but not all studies found this trend^34–37^]. Analysis of the trajectory of symptoms over time showed that most individuals were resilient or recovered within the first six months of the pandemic^26–28,30^. In contrast, another group of individuals showed sustained poor mental health or deterioration over time, suggesting that they may be at risk in the aftermath^26,30^.

It is thought that the impact of the pandemic may be more significant in developing countries as social inequalities increase, vaccination is delayed and lockdowns are extended^38^. Particularly Argentina had one of the strictest and longest lockdowns in the world. Despite most of the activities being suspended or reduced, the number of new cases grew steadily, and Argentina became one of the countries with the highest rate of infections per inhabitant (Supplementary Material Fig. S1). Previous cross-sectional reports found an initial mental health worsening similar to other countries (i.e. higher rates of psychological distress, anxiety, depression, etc.) and that distress-symptoms were best clustered by severity instead of types^7,13^. No previous study monitored mental health change in time in developing countries or periods longer than 6 months in other locations.

Here, we used a longitudinal sample (n = 832) tracked every 3–5 months since the initial Argentinian lockdown, with the overall aim of analyzing mental health trajectories during 16 months of the pandemic (April 2020–August 2021). More specifically, we aimed to identify distinct psychological distress trajectories over time and characterize associated risk/protective factors.

## Method

### Participants

We collected data on a convenience sample of 916 Argentine volunteers ranging from 18 to 90 years using an online questionnaire. Participants were recruited using social media, institutional emails, and announcements. Data collection started on April 2020 (*Time 1*), 11 days after the beginning of mandatory quarantine, continued on July 2020 (*Time 2*), October 2020 (*Time 3*), March 2021 (*Time 4*), and was completed on August 2021 (*Time 5*). When any of the participants did not complete one or more of the time points, they were excluded from the analysis. In Time 5, the final sample comprised n = 832 participants, representing an attrition rate of 9.2%. Participants did not receive any compensation for their participation. The authors assert that all procedures contributing to this work comply with the ethical standards of the relevant national and institutional committees on human experimentation and with the Helsinki Declaration of 1975, as revised in 2008. Furthermore, all procedures were approved by the FLENI ethical committee. Online informed consent was obtained from all subjects.

### Instruments

#### Psychological distress

The 53 item version (range 0–4) of the Brief Symptom Inventory (BSI-53^39^) was used to assess general mental health along its 9 symptomatic dimensions (Somatization, Anxiety, Phobic Anxiety, Obsession-Compulsion, Interpersonal Sensitivity, Depression, Hostility, Paranoid Ideation, and Psychoticism). Psychological distress was estimated according to the Global Severity Index (GSI) which corresponds to the mean score of each dimension of the BSI-53 and four additional items. The BSI-53 has been used in various psychiatric and natural settings^39–41^. It has a 9-factor structure^39,41,42^ with robust reliability (α = 0.88). However, this study did not use a representative sample or random population and therefore prevalence levels of GSI or the symptoms dimensions are not presented.

#### Trait-measures

Big Five Inventory-10^43^ was used to assess the big five personality traits: Extroversion, Agreeableness, Openness, Neuroticism, and Conscientiousness. The BFI-10 has a similar structure to the full BFI with acceptable psychometric properties (α = 0.85^44^. BFI-10 employs two items for each trait on a 1–5 Likert scale. Trait-resilience was measured with the 10-item (range 0–4) self-rated Connor-Davidson Resilience Scale^45^. It has a one-factor solution, good reliability (α = 0.91) and validity in non-clinical and clinical samples^46^.

#### State-measures

COVID-19 related fear (8-items) and Coping skills during the pandemic (5-items), were assessed using two short scales developed in a previous study^13^. Both scales consisted of a 0–4 Likert scale. The COVID-19 related fear scale measures fear of being infected, anxious appraisals about the virus and its potential consequences. On the other hand, the Coping skills during the pandemic scale, assess the ability to cope and tolerate social distancing and lockdowns. In our previous study^13^, we performed a Confirmatory Factor Analysis on the COVID-19 related fear and Coping Skills items and found a two-factor solution with acceptable reliability indices (α = 0.89 and α = 0.79).

### Sociodemographic data and covariates

Self-reported sociodemographic data were obtained, including age, gender, occupation, education level, marital status, and income level (Supplementary Material Table S1). Additional covariates were also evaluated: belonging to a known risk group for COVID-19 (yes/no), being an essential service worker (yes/no), having economic concern derived from COVID-19 (range 1–5), the overall number of hygiene measures against COVID-19 (range 1–5), time spent in COVID-19 related information and news (media exposure, range 1–5), importance given to COVID-19 related information and news (media valuation, range 1–5), exercise (yes/no), religiosity (yes/no), tobacco use (yes/no), alcohol consumption (yes/no), being previously exposed to trauma (yes/no) or diagnosed with a neurological or psychiatric disorder (yes/no). Finally, Social Network Size/Strength was measured using the Lubben Social Network Scale^47^, consisting of 12 items on a 7-point scale. This scale has shown robust reliability (α = 0.89).

### Procedure

Participants completed all sociodemographic and trait-measures at Time 1 (April 2020) along the state-measures and symptom measures (see below). Between Time 2 and Time 5, participants responded to the same questionnaire which comprised the state-measures (COVID-19 related Fear and Coping Skills scales) and symptom measures (BSI-53) as before, with the inclusion of questions regarding changes in income, media exposure and valuation, hygiene measures, work, having economic concern and being infected.

### Statistical analysis

Data analysis was implemented in R, 4.0.5 (R Foundation). When appropriate, categorical and normally distributed variables were analyzed through chi-square tests and ANOVA. Non-normally distributed variables were analyzed with Mann–Whitney-U and Kruskal–Wallis tests.

We first analyzed overall changes in Psychological Distress (GSI) along with Somatization, Anxiety, Phobic Anxiety, Obsession-Compulsion, Interpersonal Sensitivity, Depression, Hostility, Paranoid Ideation, and Psychoticism in Time (April 2020 to August 2021) including different predictors, using hierarchical mixed-effects models. Hierarchical models were implemented using the *lme4* package. We used different models that varied in complexity and number of fixed effects for each mental health outcome to evaluate its importance, using participants’ ID as a random effect. Model comparison was based on comparing models of different complexity using a Likelihood Ratio Test. When models did not have a significant difference, we then favored less complex models with lower Bayesian Information Criterion (BIC) values. In addition, we reported marginal R^2^ and conditional R^2^ as effect sizes for the winning models. Marginal R^2^ describes the variance explained by the fixed factors while conditional R^2^ indicates the proportion of variance explained by the entire model (both the fixed and random factors). Then, we used the Psychological Distress scores (GSI) to construct latent class mixture models (Growth Mixture Models—GMM) and to identify the optimal number of distinct trajectories. GSI was selected to estimate latent classes as it represents an overall index of psychological distress^42^. GMM was implemented in the *lcmm* package. We used a forward approach, starting from simple models (one-class) to more complex ones (six-class). Moreover, to improve interpretability, age and gender were included as covariates in trajectory analysis. Model fit was assessed using the BIC and entropy levels. After model selection, participants were classified according to their most likely group/trajectory. Finally, to determine potential risk/protective factors associated with each class/trajectory, all covariates and trait/state measures were entered into a mixed-effects multinomial logistic regression. Regression coefficients are presented as odds ratios (ORs) and 95% Cis.

## Results

**Figure 1:**
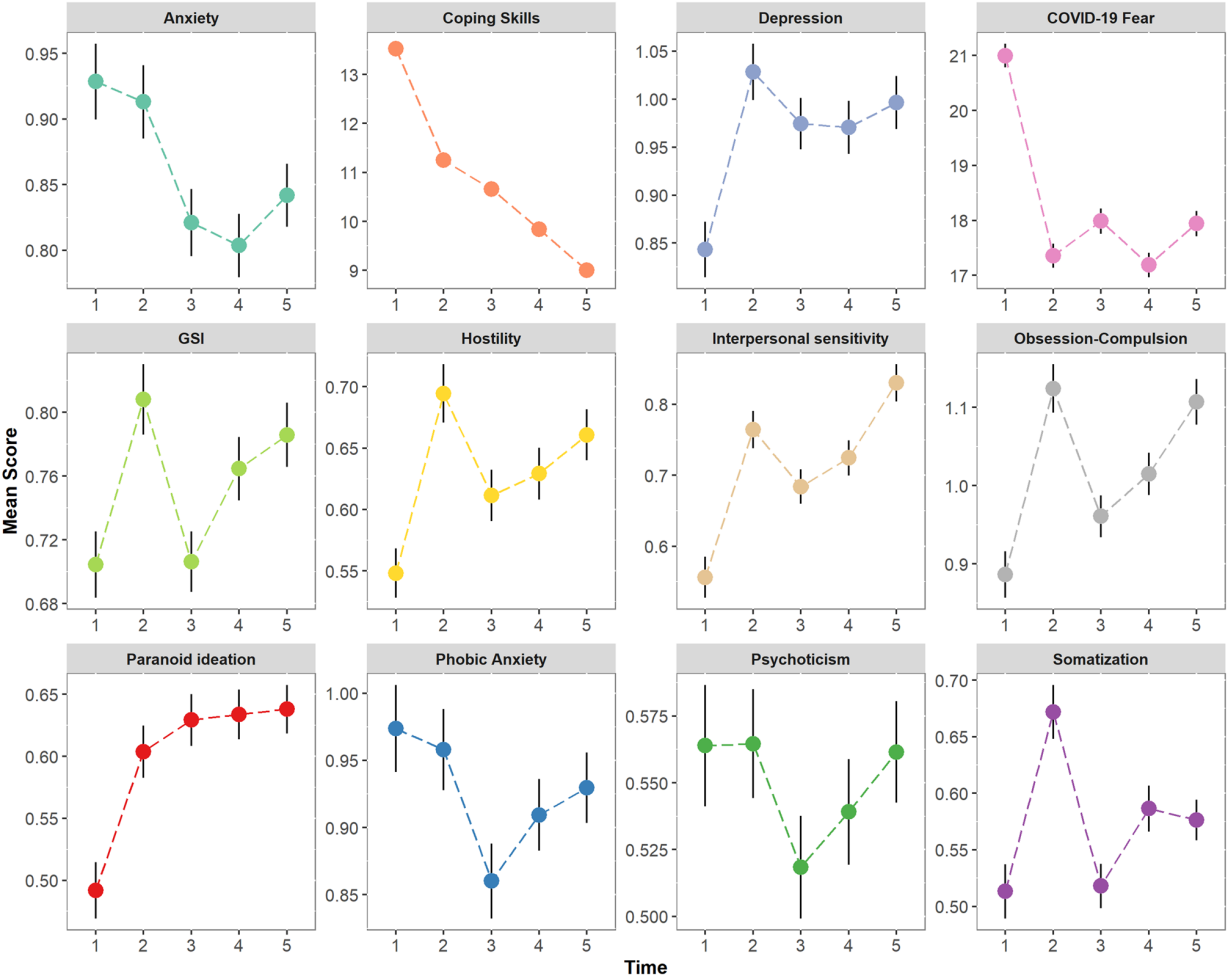
Overall mean scores of Psychological Distress (GSI), symptom dimensions of the BSI-53, and state-pandemic measures (COVID-19 related Fear and Coping skills during the pandemic scales) over Time (April 2020 to August 2021). Error bars represent standard errors.

On average, participants were aged 49.8 (SD = 16.4) years old, most of them were women (79.8%), and 24.5% had a previous neuropsychiatric diagnosis (Table S1). Analysis of mean scores over time of Psychological Distress (GSI) and the BSI symptom dimensions revealed to be heterogeneous, as some aspects of mental health improved and others declined (Fig. 1). Model comparison of each symptom dimension revealed that models which included age, gender, and being previously diagnosed with a neuropsychiatric disorder, were those with the better model fit (see Supplementary Material for details). Notably, the inclusion of changes in income, media exposure and valuation, hygiene measures, work changes, and having economic concerns across time, did not improve model fit. In general, individuals who are younger, female, or have pre-existing psychiatric diagnoses reported higher levels of psychological distress (see Supplementary Material Figs. S2–S4). Overall, psychological distress increased over time (β = 0.012, p = 0.032, R^2^_Marginal_ = 0.09, R^2^_Conditional=_0.47) whereas fear-related symptoms such as Anxiety (β = − 0.028, p < 0.001, R^2^_Marginal_ = 0.08, R^2^_Conditional_ = 0.43) and COVID-19 related Fear (β = − 3.78, p < 0.001, R^2^_Marginal_ = 0.06, R^2^_Conditional_ = 0.39) tended to decrease. In contrast to other reports^27,28^, we found that Depression (β = 0.025, p = 0.002, R^2^_Marginal_ = 0.10, R^2^_Conditional_ = 0.45), Hostility (β = 0.016, p = 0.006, R^2^_Marginal_ = 0.09, R^2^_Conditional_ = 0.38), Interpersonal Sensitivity (β = 0.051, p < 0.001, R^2^_Marginal_ = 0.12, R^2^_Conditional_ = 0.44), Obsession-Compulsion (β = 0.032, p < 0.001, R^2^_Marginal_ = 0.08, R^2^_Conditional_ = 0.43) and Paranoid-Ideation (β = 0.032, p < 0.001, R^2^_Marginal_ = 0.07, R^2^_Conditional_ = 0.39) levels significantly increased over time. The highest symptom levels were observed at Time 2 (July 2020) after 3 months of continuous strict quarantine, and Time 5 (August 2021) 2 months after the second wave peaked (Supplementary Material Fig. S1). Finally, Phobic Anxiety (β = − 0.014, p = 0.109, R^2^_Marginal_ = 0.05, R^2^_Conditional_ = 0.39), Somatization (β = 0.010, p = 0.102, R^2^_Marginal_ = 0.05, R^2^_Conditional_ = 0.40), Psychoticism (β = − 0.003, p = 0.594, R^2^_Marginal_ = 0.06, R^2^_Conditional_ = 0.40) levels remained relatively stable (see Supplementary Material Table S2 for full details).

We then fitted different Growth Mixture Models (GMM) based on the GSI, with one to six latent classes to identify distinct psychological distress trajectories over time. We considered the four-class solution the best-fitting model as it provides lower BIC values than the one-to-three class solution and it is more parsimonious than the five or six-class solution (see Supplementary Material Table S3). The five and six-class solution have the lower BIC values; however, they contain inadmissible sample sizes (< 5% of the entire sample^48^; Model details of the chosen four-class solution can be found in the Supplementary Material Table S4).

**Figure 2:**
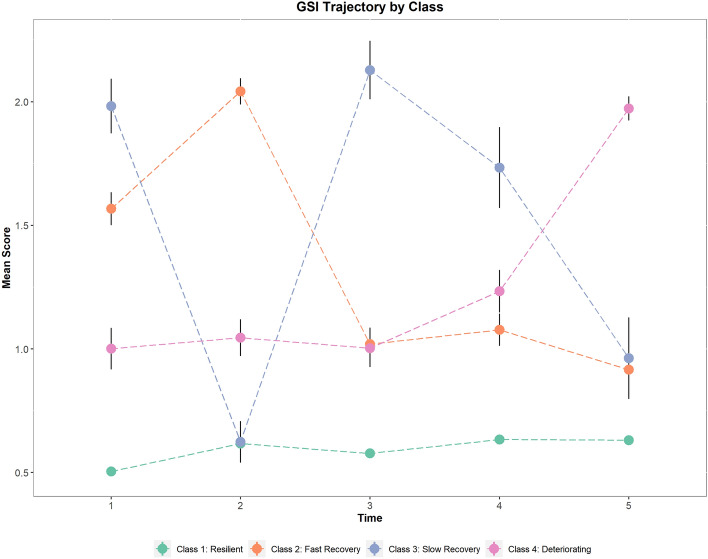
Estimated mean Psychological Distress (GSI) score from the four-class solution of the Growth Mixture Model over time. Each class indicates a specific trajectory during the pandemic. Error bars represent standard errors.

Figure 2 shows the psychological distress (GSI) trajectories over time from the four-class model. Most participants had a consistently good or resilient mental health (Class 1, Resilient = 73.9% participants) from the start of the mandatory lockdown (April 2020–Time 1; M = 0.50, SD = 0.36) to August 2021 (Time 5, M = 0.63, SD = 0.41), where preventive measure were at their minimum and the vaccination campaign peaked. A Fast Recovery group (Class 2 = 10.8% participants) demonstrated mental health worsening from April 2020 (Time 1, M = 1.57, SD = 0.62) to July 2020 (Time 2, M = 2.05, SD = 0.5), then Psychological Distress levels decreased from October 2020 (Time 3, M = 1.02, SD = 0.63) to August 2021 (Time 5, M = 0.92, SD = 0.54). In contrast, a Slow Recovery group (Class 3 = 6.7% participants) improved their Psychological Distress from April 2020 (Time 1, M = 1.98, SD = 0.54) to July 2020 (Time 2, M = 0.62, SD = 0.40) but showed a deterioration in October 2020 (Time 3, M = 2.13, SD = 0.56). This class “recovered” in August 2021 (Time 5, M = 0.96, SD = 0.76), as it exhibited their lowest Psychological Distress levels, which were similar to those observed in the Fast recovery class. Finally, we detected a Deteriorating group (Class 4, 8.5% participants), which showed stable levels of Psychological Distress from April 2020 (Time 1, M = 1.05, SD = 0.60) to October 2020 (Time 3, M = 1, SD = 0.64) but then mental health declined steadily from March 2021 (Time 4, M = 1.27, SD = 0.70) to August 2021 (Time 5, M = 1.99, SD = 0.40). Analysis of participant’s characteristics showed that age, income level, being previously exposed to a trauma or being previously diagnosed with a neuropsychiatric disorder were significantly different between classes/trajectories (Table 1).

**Table 1:** Sociodemographic characteristics, covariates, psychological distress, personality and resilience scores by class/trajectory.

	Class 1	Class 2	Class 3	Class 4	Total	p value
	Resilient	Fast recovery	Slow recovery	Deteriorating		
Overall (no. %)	615 (73.9%)	90 (10.8%)	56 (6.7%)	71 (8.5%)	832 (100%)	
**Age range**						< 0.001
18–29	44 (8.0%)	16 (17.8%)	12 (16.7%)	14 (19.7%)	86 (10.3%)	
30–44	153 (24.9%)	28 (31.1%)	12 (16.7%)	20 (28.2%)	213 (25.6%)	
45–64	257 (41.0%)	43 (47.8%)	19 (45.8%)	31 (43.7%)	350 (42.1%)	
> 65	161 (26.1%)	3 (3.3%)	13 (20.8%)	6 (8.5%)	183 (22.0%)	
**Gender**						0.071
Men	126 (21.9%)	11 (12.2%)	18 (8.3%)	13 (18.3%)	168 (20.2%)	
Women	489 (78.1%)	79 (87.8%)	38 (91.7%)	58 (81.7%)	664 (79.8%)	
**Essential service worker**						0.513
No	521 (83.0%)	80 (88.9%)	37 (87.5%)	60 (84.5%)	698 (83.9%)	
Yes	94 (17.0%)	10 (11.1%)	19 (12.5%)	11 (15.5%)	134 (16.1%)	
**Education level**						0.078
Low	543 (86.4%)	70 (77.8%)	35 (79.2%)	54 (76.1%)	702 (84.4%)	
Middle	7 (1.1%)	3 (3.3%)	0 (0.0%)	2 (2.8%)	12 (1.4%)	
High	65 (12.5%)	17 (18.9%)	21 (20.8%)	15 (21.1%)	118 (14.2%)	
**Marital status**						0.444
Divorced	88 (14.8%)	14 (15.6%)	11 (12.5%)	7 (9.9%)	120 (14.4%)	
Married	311 (49.3%)	40 (44.4%)	17 (37.5%)	34 (47.9%)	402 (48.3%)	
Unmarried	184 (29.7%)	33 (36.7%)	19 (45.8%)	28 (39.4%)	264 (31.7%)	
Widow/er	32 (6.2%)	3 (3.3%)	9 (4.2%)	2 (2.8%)	46 (5.5%)	
**Income level**						0.026
Lower	86 (14.5%)	13 (14.4%)	17 (37.5%)	9 (12.7%)	125 (15.0%)	
Middle	236 (37.7%)	44 (48.9%)	19 (45.8%)	28 (39.4%)	327 (39.3%)	
Upper	103 (17.2%)	10 (11.1%)	10 (8.3%)	14 (19.7%)	137 (16.5%)	
Upper_Middle	190 (30.6%)	23 (25.6%)	10 (8.3%)	20 (28.2%)	243 (29.2%)	
**Ocuppation**						< 0.001
Employed	270 (42.8%)	43 (47.8%)	15 (33.3%)	32 (45.1%)	360 (43.3%)	
House wife	20 (3.9%)	7 (7.8%)	8 (4.2%)	8 (11.3%)	41 (4.9%)	
Retiree	186 (29.8%)	10 (11.1%)	12 (29.2%)	10 (14.1%)	220 (26.4%)	
Self employed	102 (16.5%)	17 (18.9%)	7 (8.3%)	12 (16.9%)	138 (16.6%)	
Student	22 (4.2%)	7 (7.8%)	9 (16.7%)	8 (11.3%)	46 (5.5%)	
Unemployed	13 (2.8%)	6 (6.7%)	7 (8.3%)	1 (1.4%)	27 (3.2%)	
**Pertains to risk group**						0.183
No	329 (53.3%)	58 (64.4%)	28 (50.0%)	35 (49.3%)	450 (54.1%)	
Yes	286 (46.7%)	32 (35.6%)	28 (50.0%)	36 (50.7%)	382 (45.9%)	
**Exercise**						0.003
No	263 (43.1%)	55 (61.1%)	30 (58.3%)	39 (54.9%)	387 (46.5%)	
Yes	368 (56.9%)	35 (38.9%)	26 (41.7%)	32 (45.1%)	445 (53.5%)	
**Religious**						0.977
No	261 (40.3%)	37 (41.1%)	25 (37.5%)	30 (42.3%)	337 (40.5%)	
Yes	352 (59.7%)	53 (58.9%)	31 (62.5%)	41 (57.7%)	495 (59.5%)	
**Previous trauma**						0.009
No	449 (71.9%)	53 (58.9%)	28 (50.0%)	53 (74.6%)	583 (70.1%)	
Yes	166 (28.1%)	37 (41.1%)	28 (50.0%)	18 (25.4%)	249 (29.9%)	
**Tobacco**						0.589
No	534 (85.0%)	73 (81.1%)	38 (91.7%)	61 (85.9%)	706 (84.9%)	
Yes	81 (15.0%)	17 (18.9%)	18 (8.3%)	10 (14.1%)	126 (15.1%)	
**Alcohol**						0.314
No	282 (46.1%)	49 (54.4%)	30 (58.3%)	32 (45.1%)	393 (47.2%)	
Yes	333 (53.9%)	41 (45.6%)	26 (41.7%)	39 (54.9%)	439 (52.8%)	
**Diagnosed**						< 0.001
No	514 (79.4%)	50 (55.6%)	28 (50.0%)	52 (73.2%)	628 (75.5%)	
Yes	133 (20.6%)	40 (44.4%)	28 (50.0%)	19 (26.8%)	204 (24.5%)	
Extroversion	2.780 (0.749)	2.817 (0.925)	2.688 (0.832)	2.859 (0.816)	2.788 (0.777)	0.756
Agreeableness	2.908 (0.695)	3.111 (0.806)	2.812 (0.656)	3.141 (0.633)	2.947 (0.706)	0.004
Conscientiousness	1.910 (0.762)	2.256 (0.839)	2.521 (1.184)	2.268 (0.788)	1.995 (0.803)	< 0.001
Neuroticism	3.509 (0.539)	3.883 (0.586)	4.083 (0.620)	3.669 (0.534)	3.580 (0.565)	< 0.001
Opennes	2.242 (0.864)	2.256 (0.934)	2.292 (0.966)	2.373 (0.740)	2.256 (0.864)	0.678
Resilience	29.906 (5.987)	24.300 (7.936)	23.375 (8.085)	27.704 (5.979)	28.923 (6.596)	< 0.001
Social network size	35.453 (8.390)	31.400 (11.805)	30.696 (9.068)	31.778 (8.174)	34.593 (8.892)	< 0.001

**Figure 3:**
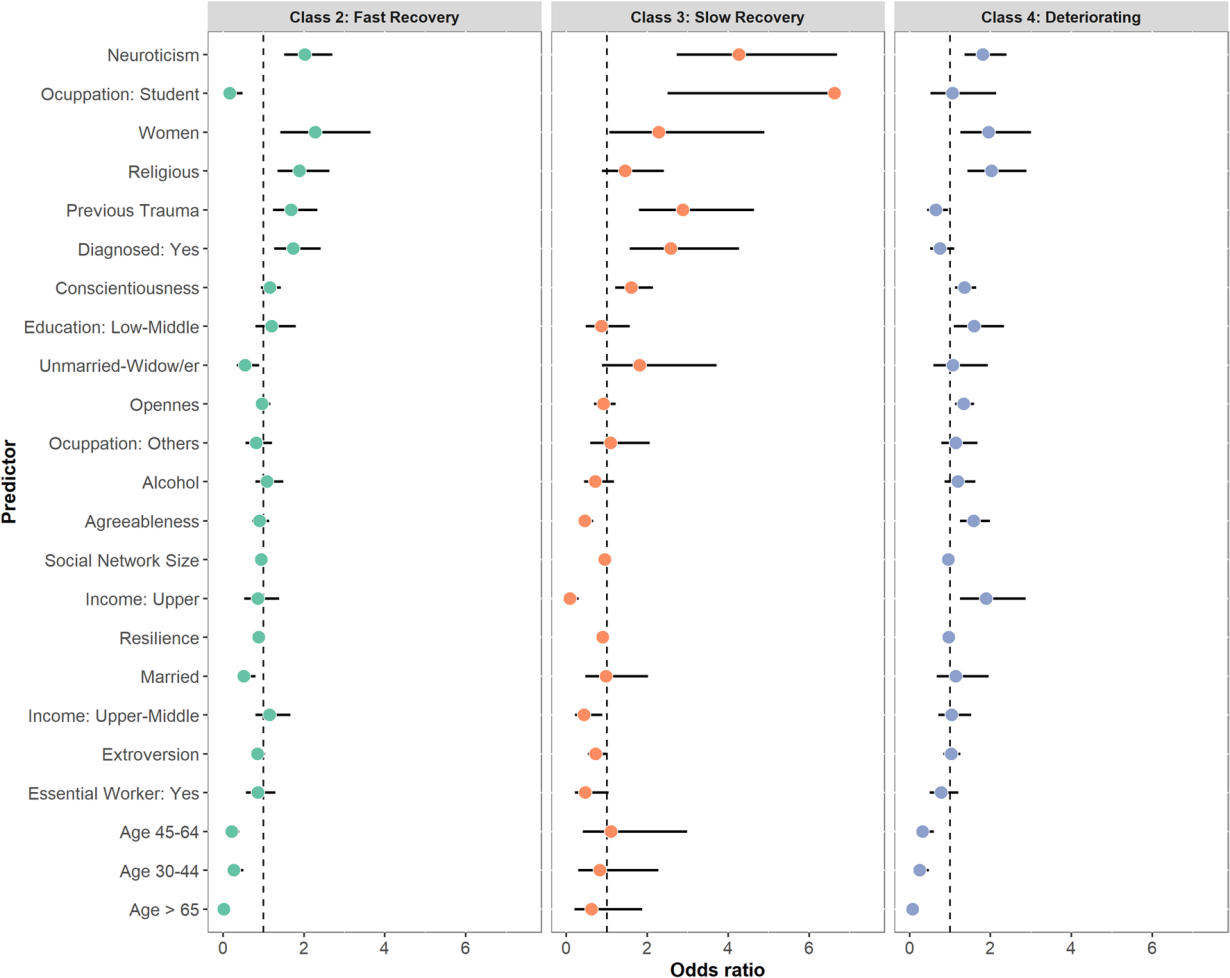
Risk and protective factors for Psychological Distress associated with trajectories by multinomial Logistic regression. Class 1 (Resilient) served as reference. Results are expressed as odds ratio with 95% CI. Due to their small size, the following categories were combined for analysis: Education: middle and high levels were combined into middle-high level; Marriage status: Unmarried and Widow/er were combined into Unmarried-Widow/er; Income: Lower and middle were combined into Lower-Middle; Employment status: Retiree, House Wife and Unemployed were combined into “Others”.

To determine risk and protective factors associated with class membership, we performed a multinomial logistic regression, using the Resilient class (Class 1) as reference (Fig. 3; see Supplementary Material Table S5 for details). We found that classes with no consistently good mental health (Fast/Slow Recovery and Deterioration trajectories) were associated with higher scores in trait neuroticism and gender (women). Trait-Resilience and Social-Network size were protective factors against fluctuating (Fast/Slow Recovery classes) or declining (Deteriorating class) psychological distress over time. Moreover, having a previous psychiatric/neurological diagnosis and being previously exposed to trauma were positively associated with Fast Recovery and Slow recovery trajectories. In contrast, adults (30–65 years) and older adults (> 65 years) were associated with lesser odds of being in the Fast Recovery and Deteriorating groups. Sociodemographic variables such as high-income level, were specific predictors of the Deteriorating class. In addition, higher scores of Agreeableness, Conscientiousness, and Openness were associated with a decline of mental health in time (Deteriorating class).

**Figure 4:**
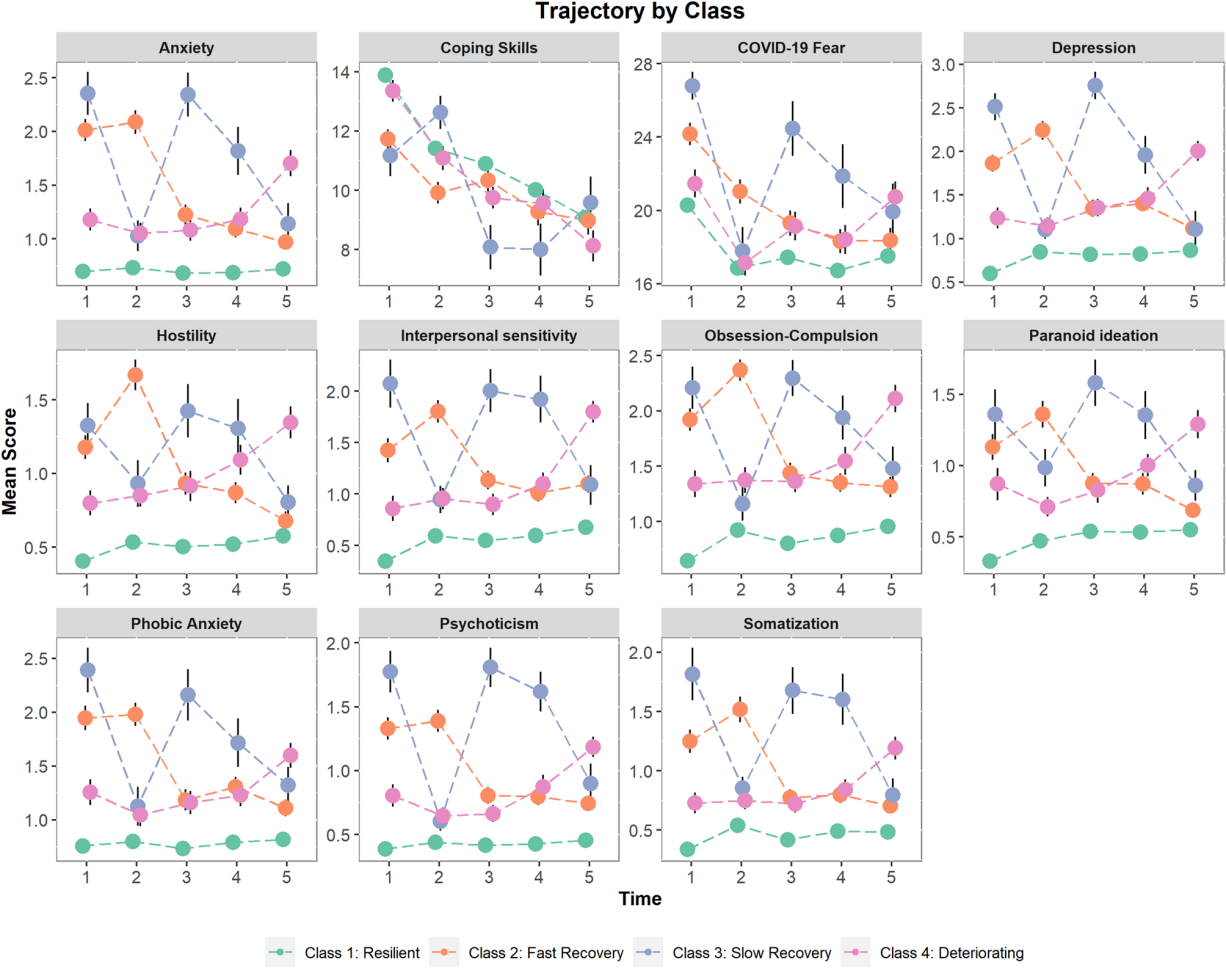
Estimated mean scores for each symptom dimension (BSI-53) and state-measures from the four-class solution of the Growth Mixture Model (GMM) over time. Class membership was estimated previously from Psychological Distress (GSI) scores. Error bars represent standard errors.

Finally, participants´ most likely latent class membership from the GMM analysis was used to analyze each symptom dimension over time (Fig. 4). That is to say, each of the four class membership was used to analyze the trajectory of the symptom dimension over the 5 time points. As expected, symptom trajectories based on latent classes revealed similar trends as those observed in Psychological Distress trajectories. That is: (1) participants in the Resilient group (Class 1) showed relatively stable low scores over time; (2) participants in the Fast Recovery group (Class 2) had symptom improvement between Time 1 (April 2020) and Time 3 (October 2020); (3) participants in the Slow recovery group (Class 3), demonstrated a decrease in symptoms only after 16 months of the pandemic (Time 5—August 2021); (4) participants in the Deteriorating group (Class 4) had a stable mild-moderate symptom level across time, until Time 5 (August 2021) where they peaked. In addition, changes in work, income level, or having economic concern over time did not show a distinct trajectory as a function of class membership (Supplementary Material Fig. S5).

## Discussion

In the present study on 832 Argentinian participants, we examined Psychological Distress temporal dynamics across 16 months (April 2020–August 2021) and several symptom dimensions associated with the COVID-19 pandemic. Mental health was revealed to be heterogeneous as fear-related symptoms (COVID-19 fear and Anxiety levels) decreased following the introduction of a strict lockdown, whereas mood symptoms, negative affect, difficulties in social relationship and regulating impulses, tended to increase (Depression, Hostility, Interpersonal Sensitivity, Obsession-Compulsion, and Paranoid-Ideation). However, as the observed effect sizes (R^2^) are relatively small, these effects should be interpreted cautiously and take into account the wide variability of the pandemic experience between subjects. Furthermore, we identified that being young, women or having a previous neuropsychiatric diagnosis were predictors of more intense psychological distress and related-symptoms over time. Previous longitudinal studies mostly focused on anxiety and depression symptoms and reported an opposite pattern, which indicated that overall mental health improved months after lockdown^26–32^. These data come from more developed countries (i.e. Germany, England, USA, China) where lockdowns and preventive measures were shorter or more flexible compared to Argentina. Moreover, the economic and social cost between countries is radically different. However, other longitudinal research showed mental health worsening during the first months of the COVID-19 outbreak^34–37^, suggesting that the relation between the pandemic and Psychological Distress is not uniform but rather a complex phenomenon.

Despite finding that mental health deteriorated during the pandemic, the heterogeneous course of symptoms over time may bias the results and obscure the existence of subgroups in the sample. Therefore, trajectory analysis allowed us to analyze the complexity and specificity of the impact of the pandemic among the different individuals. Using latent class analysis, we identified the existence of four distinct symptom trajectories: (1) The Resilient trajectory, which compromised the majority of participants (73.9%), had a consistently good mental health throughout the pandemic as their psychological distress levels and related symptoms were the lowest and stable over time; (2) The Fast Recovery trajectory (10.8%) had a substantial improvement in Psychological distress levels 6 months after the mandatory lockdown (October 2020) where restrictive measures began to relax, and continued to recover until August 2021; (3) The Slow Recovery trajectory (6.7%) displayed a rebound in Psychological distress levels between April 2020–October 2020, when the first wave peaked and showed better mental health following 10 months (August 2021); (4) The Deteriorating trajectory (8.5%) maintained a mild level of Psychological Distress from April 2020 to October 2020 and began to deteriorate in March 2021 until August 2021 where symptoms levels reached their peak. Several circumstances might be related to the improvement of mental health in the Slow/Fast recovery classes over time. For example, relaxation of restrictive measures, the start of the vaccination campaign, outside activity permissions, and school re-openings in some districts. In addition, mental health improvement over time may indicate a process of adaptation to long stressful events that promote the emergence of new coping skills^25,49^.

Our study described potential risk and protective factors associated with distinct Psychological Distress trajectories. First, individual characteristics such as being young (< 30 years), women, having a smaller social-network size, having a psychiatric/neurological disorder, or being previously exposed to trauma, were associated with an initial increase in Psychological Distress and related symptoms, after the mandatory quarantine (April 2020), followed by a Fast/Slow improvement over time (Fast Recovery and Slow Recovery trajectories). Accordingly, having upper-middle and upper-income levels, were specifically associated with mental health worsening over time (Deteriorating trajectory). Second, following previous cross-sectional reports, we provided evidence that trait-characteristic are differentially linked to mental health outcomes^13,18,20^. In this sense, higher levels of neuroticism and lower levels of resilience were associated with higher odds of being in the non-resilient trajectories, and Agreeableness, Conscientiousness, and Openness were specifically related to the Deteriorating trajectory. These results may seem unusual given that high Neuroticism is a strong predictor of worse mental health outcomes, but high Conscientiousness, and Openness, are associated with better outcomes^50^. However, a recent longitudinal study performed in the COVID-19 period found also that high Openness, Agreeableness and Conscientiousness predicted mental health deterioration over time^51^. These results could be understood by considering how the pandemic restrictions affected these behavioral traits. Lockdowns, social distancing, and other economic restrictions, limited the opportunity to engage in new experiences (Openness trait), fulfill altruistic, and social needs (Agreeableness trait), and maintain motivational stability and the fulfillment of responsibilities (Conscientiousness trait). Current findings are in line with other longitudinal reports during the COVID-19 pandemic which found Resilient, Recovered, and Deteriorating trajectories, associated with similar sociodemographic risk factors^26,28^.

The present study has several limitations. Our sample was unrepresentative of the population which may have compromised the observed effect sizes. Most of our respondents were women and participants with middle-to-upper income levels, which may have obscured the relation between economic variables (income and work-related changes over time) and mental health trajectories. In addition, despite the economic efforts made by the Argentinian government, the poverty rate in the first semester of 2021 was 40.9%, which represented approximately a 10% increase compared to pre-pandemic levels (National Institute of Statistics and Census of Argentina—INDEC). Another important limitation is the absence of baseline or pre-pandemic mental health measures that would have facilitated the interpretation of the symptoms trajectories.

To the best of our knowledge, this is the first study to explore the pandemic effects in mental health over a long period of time (> 6 months), and more specifically, in a developing country. With its social and economic inequalities, Argentina had one of the longest and strictest lockdowns in the world, which may have contributed to the observed differences between this study and others performed in more developed countries. Overall, we found that mental health trajectory during the COVID-19 pandemic had a complex and heterogeneous dynamic, suggesting that the pandemic experience was different in each individual. At first sight, Psychological Distress levels and related symptoms increased as the pandemic in Argentina unfolded (April 2020–August 2021). However, a careful latent class analysis enabled the characterization of distinct mental health trajectories with their associated risk/protective factors. Finally, this study helped to identify a vulnerable subgroup of individuals with deteriorating mental health, which may need professional assistance. Preventive interventions might be useful to target this group at risk and improve their mental health.

## Supplementary Information

Supplementary Information.

## Data Availability

The datasets obtained in the current study are available from the corresponding author upon request.
