# Supplementary material for: Psychological distress and mental health trajectories during the COVID-19 pandemic in Argentina: a longitudinal study
Source: Sci Rep. 2022 Apr 4;12:5632. doi: 10.1038/s41598-022-09663-2 (PMC8979149; doi:10.1038/s41598-022-09663-2)

**Supplementary Material**

*Psychological Distress and mental health trajectories during the COVID-19 pandemic in Argentina: a longitudinal study.*

**Figure S1. COVID-19 in Argentina**

**
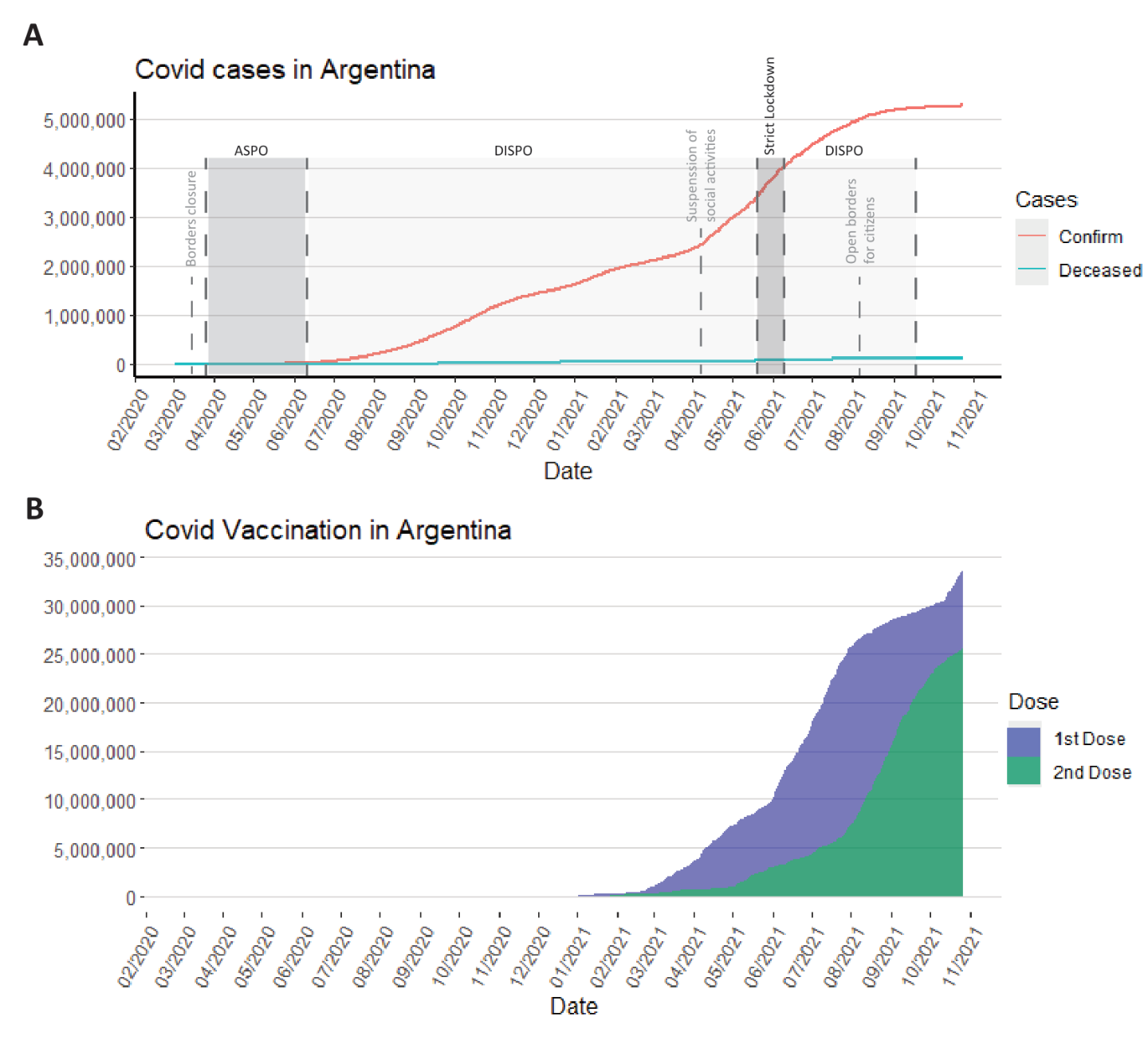
**

**Figure S1.** (A) Covid Cases in Argentina. Government measures of total and mandatory quarantine “ASPO” (preventive and compulsory social isolation); Social, preventive, and compulsory distancing was called “DISPO”. (B) Covid Vaccination in Argentina.

**Table S1.** Sociodemographic and Trait-Measures in the sample.

|  | **Overall (N=832)** |
| --- | --- |
| **Age Range** |  |
| 18-29 | 86 (10.3%) |
| 30-44 | 213 (25.6%) |
| 45-64 | 350 (42.1%) |
| > 65 | 183 (22.0%) |
| **Gender** |  |
| - Men | 168 (20.2%) |
| - Women | 664 (79.8%) |
| **Essential Service Worker** |  |
| - No | 698 (83.9%) |
| - Yes | 134 (16.1%) |
| **Education Level** |  |
| - Low | 12 (1.4%) |
| - Middle | 118 (14.2%) |
| - High | 702 (84.4%) |
| **Marital Status** |  |
| - Divorced | 120 (14.4%) |
| - Married | 402 (48.3%) |
| - Unmarried | 264 (31.7%) |
| - Widow/er | 46 (5.5%) |
| **Income Level** |  |
| - Lower | 125 (15.0%) |
| - Middle | 327 (39.3%) |
| - Upper | 137 (16.5%) |
| - Upper_Middle | 243 (29.2%) |
| **Ocuppation** |  |
| - Employed | 360 (43.3%) |
| - House Wife | 41 (4.9%) |
| - Retiree | 220 (26.4%) |
| - Self Employed | 138 (16.6%) |
| - Student | 46 (5.5%) |
| - Unemployed | 27 (3.2%) |
| **Pertains to risk group** |  |
| - No | 450 (54.1%) |
| - Yes | 382 (45.9%) |
| **Exercise** |  |
| - No | 387 (46.5%) |
| - Yes | 445 (53.5%) |
| **Religious** |  |
| - No | 337 (40.5%) |
| - Yes | 495 (59.5%) |
| **Previous Trauma** |  |
| - No | 583 (70.1%) |
| - Yes | 249 (29.9%) |
| **Tobacco** |  |
| - No | 706 (84.9%) |
| - Yes | 126 (15.1%) |
| **Alcohol** |  |
| - No | 393 (47.2%) |
| - Yes | 439 (52.8%) |
| **Diagnosed** |  |
| - No | 628 (75.5%) |
| - Yes | 204 (24.5%) |
| **Extroversion** | 2.788 (0.777) |
| **Agreeableness** | 2.947 (0.706) |
| **Conscientiousness** | 1.995 (0.803) |
| **Neuroticism** | 3.580 (0.565) |
| **Opennes** | 2.256 (0.864) |
| **Resilience** | 28.923 (6.596) |
| **Social Network Size (Lubben Scale)** | 34.593 (8.892) |

*Frequency (percentage).

**Model Comparison (mixed effects analysis) of symptom dimensions (BSI-53) over Time.**

***** “Pandemic measures” refers to changes in income, media exposure and valuation, hygiene measures, work changes, having economic.

**Anxiety**

**
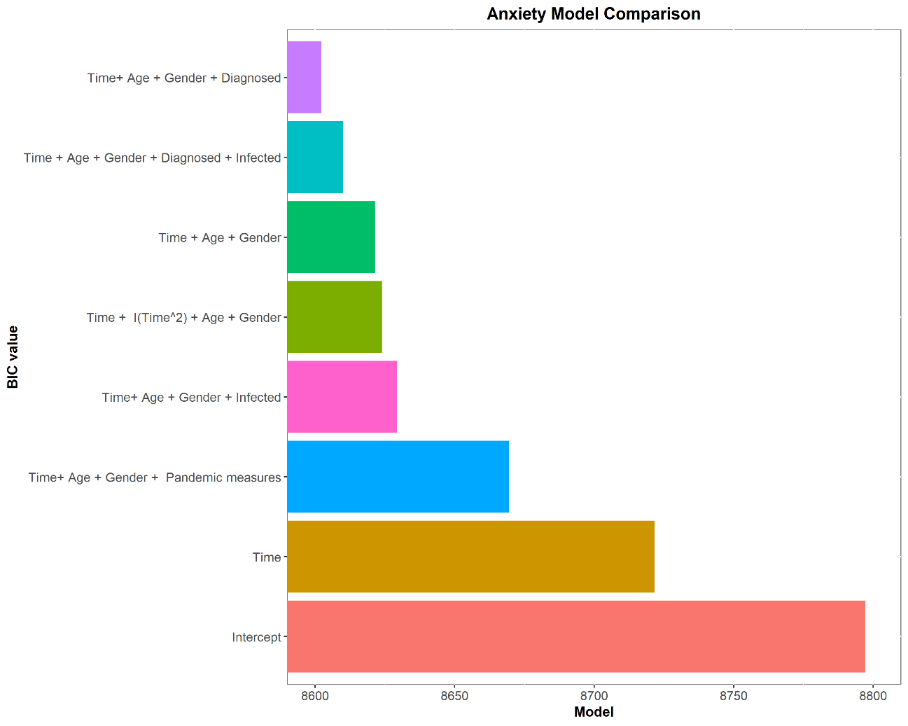
**

| **Linear mixed model: Anxiety** | | | | | |
| --- | --- | --- | --- | --- | --- |
| **Term** | **Coefficient** | **SD** | **T value** | **df** | **p.** |
| **(Intercept)** | 1.154 | 0.075 | 15.387 | 1.042.115 | 0 |
| **Time** | -0.028 | 0.007 | -3.784 | 831.000 | 0 |
| **Age** | -0.009 | 0.001 | -8.254 | 828.000 | 0 |
| **Gender: Women** | 0.235 | 0.041 | 5.738 | 828.000 | 0 |
| **Diagnosed: Yes** | 0.201 | 0.038 | 5.323 | 828.000 | 0 |
| **sd__(Intercept)** | 0.652 |  |  |  |  |
| **cor__(Intercept).Time** | -0.856 |  |  |  |  |
| **sd__Time** | 0.112 |  |  |  |  |
| **sd__Observation** | 0.582 |  |  |  |  |

**Coping Skills**

**
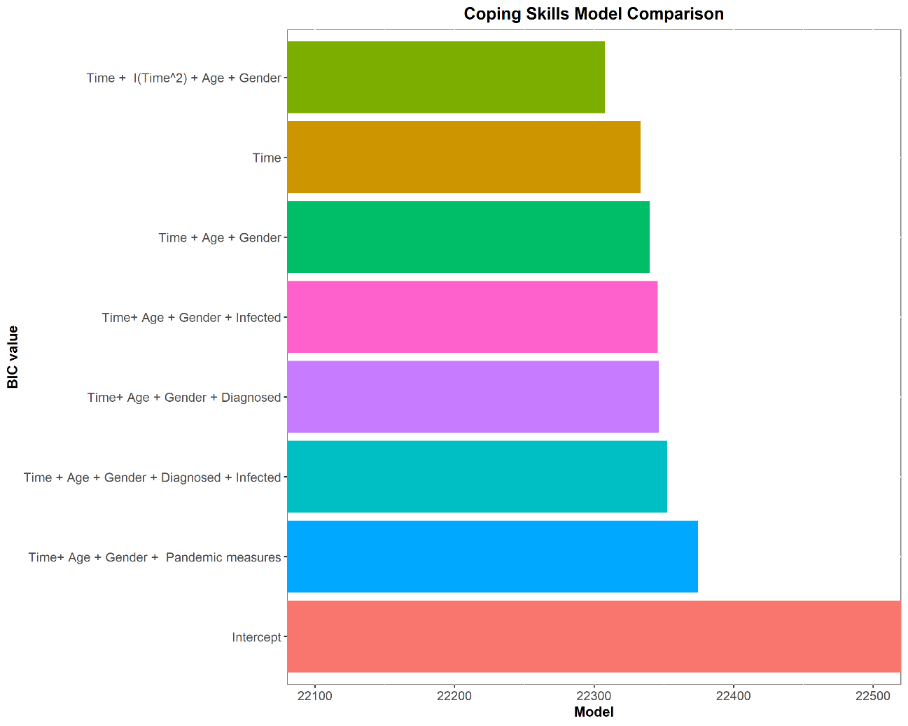
**

| **Linear mixed model: Coping Skills** | | | | | |
| --- | --- | --- | --- | --- | --- |
| **Term** | **Coefficient** | **SD** | **T value** | **df** | **p.** |
| **(Intercept)** | 14.627 | 0.386 | 37.931 | 1.823.297 | 0.000 |
| **Time** | -2.177 | 0.182 | -11.973 | 2.712.186 | 0.000 |
| **Time (Quadratic)** | 0.189 | 0.030 | 6.364 | 2.494.984 | 0.000 |
| **Age** | 0.015 | 0.005 | 3.099 | 829.000 | 0.002 |
| **Gender: Women** | -0.070 | 0.182 | -0.386 | 829.000 | 0.699 |
| **sd__(Intercept)** | 0.973 |  |  |  |  |
| **cor__(Intercept).Time** | -0.042 |  |  |  |  |
| **sd__Time** | 0.430 |  |  |  |  |
| **sd__Observation** | 3.199 |  |  |  |  |

**Depression**

**
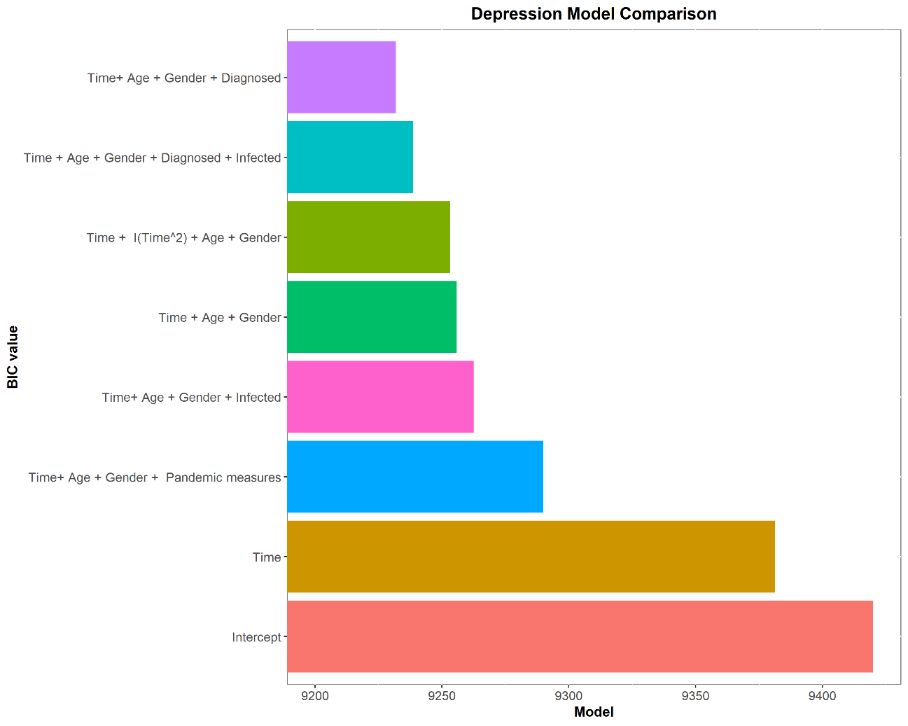
**

| **Linear mixed model: Depression** | | | | | |
| --- | --- | --- | --- | --- | --- |
| **Term** | **Coefficient** | **SD** | **T value** | **df** | **p.** |
| **(Intercept)** | 1.430 | 0.080 | 17.897 | 1.024.726 | 0.000 |
| **Time** | 0.025 | 0.008 | 3.060 | 831.000 | 0.002 |
| **Age** | -0.013 | 0.001 | -11.651 | 828.000 | 0.000 |
| **Gender: Women** | 0.092 | 0.044 | 2.091 | 828.000 | 0.037 |
| **Diagnosed: Yes** | 0.232 | 0.040 | 5.742 | 828.000 | 0.000 |
| **sd__(Intercept)** | 0.652 |  |  |  |  |
| **cor__(Intercept).Time** | -0.796 |  |  |  |  |
| **sd__Time** | 0.125 |  |  |  |  |
| **sd__Observation** | 0.627 |  |  |  |  |

**COVID-19 related Fear**

**
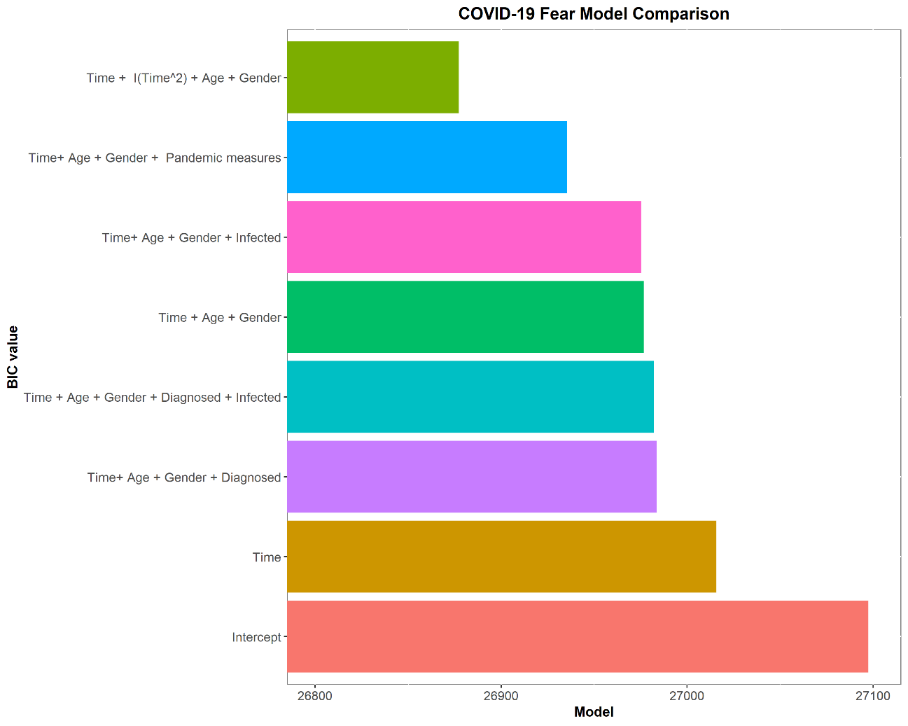
**

| **Linear mixed model: COVID-19 Fear** | | | | | |
| --- | --- | --- | --- | --- | --- |
| **Term** | **Coefficient** | **SD** | **T value** | **df** | **p.** |
| **(Intercept)** | 19.594 | 0.725 | 27.042 | 1.623.802 | 0 |
| **Time** | -3.781 | 0.307 | -12.314 | 2.705.224 | 0 |
| **Time (Quadratic)** | 0.525 | 0.050 | 10.496 | 2.494.999 | 0 |
| **Age** | 0.049 | 0.009 | 5.369 | 829.000 | 0 |
| **Gender: Women** | 2.201 | 0.355 | 6.206 | 829.000 | 0 |
| **sd__(Intercept)** | 3.955 |  |  |  |  |
| **cor__(Intercept).Time** | -0.580 |  |  |  |  |
| **sd__Time** | 0.640 |  |  |  |  |
| **sd__Observation** | 5.404 |  |  |  |  |

**Global Severity Index (GSI)**

**
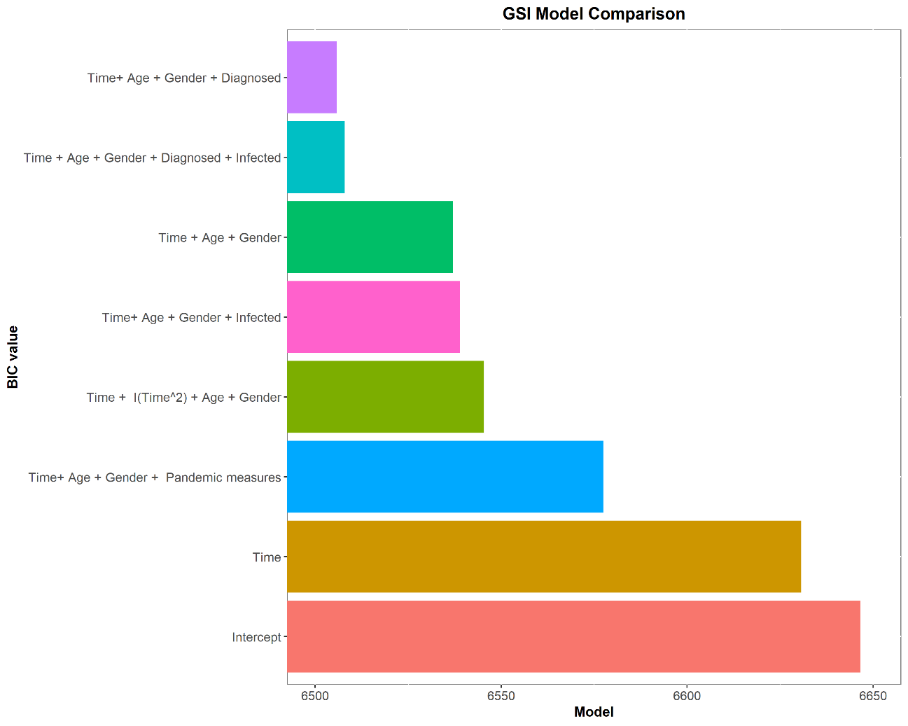
**

| **Linear mixed model: GSI** | | | | | |
| --- | --- | --- | --- | --- | --- |
| **Term** | **Coefficient** | **SD** | **T value** | **df** | **p.** |
| **(Intercept)** | 0.971 | 0.060 | 16.319 | 991.413 | 0.000 |
| **Time** | 0.012 | 0.006 | 2.153 | 831.000 | 0.032 |
| **Age** | -0.008 | 0.001 | -9.231 | 828.000 | 0.000 |
| **Gender: Women** | 0.125 | 0.033 | 3.792 | 828.000 | 0.000 |
| **Diagnosed: Yes** | 0.194 | 0.030 | 6.390 | 828.000 | 0.000 |
| **sd__(Intercept)** | 0.446 |  |  |  |  |
| **cor__(Intercept).Time** | -0.744 |  |  |  |  |
| **sd__Time** | 0.071 |  |  |  |  |
| **sd__Observation** | 0.454 |  |  |  |  |

**Hostility**

**
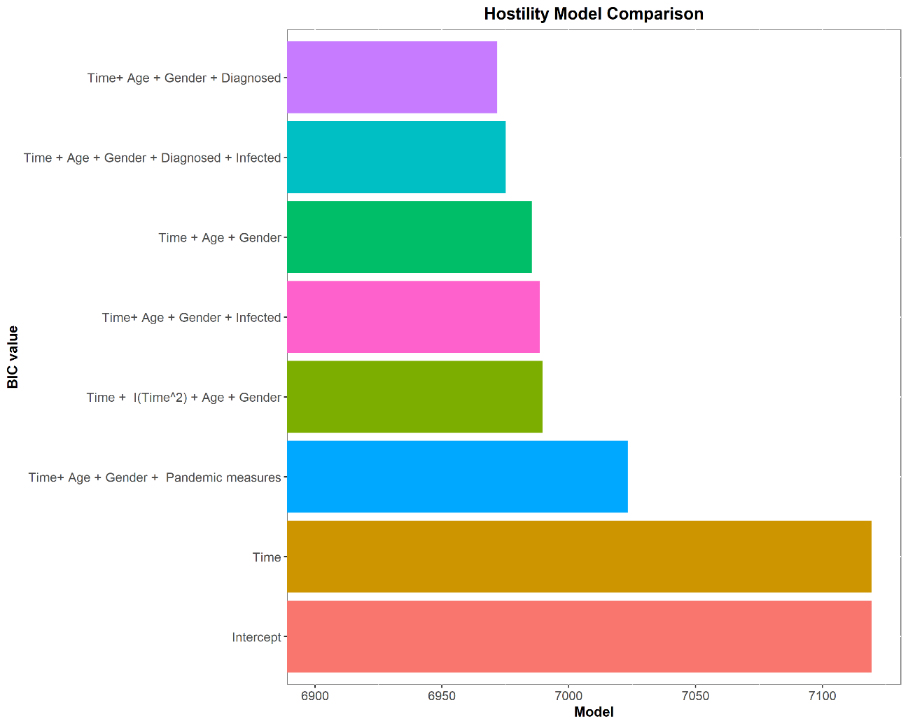
**

| **Linear mixed model: Hostility** | | | | | |
| --- | --- | --- | --- | --- | --- |
| **Term** | **Coefficient** | **SD** | **T value** | **df** | **p.** |
| **(Intercept)** | 1.119 | 0.061 | 18.478 | 989.305 | 0.000 |
| **Time** | 0.016 | 0.006 | 2.737 | 831.000 | 0.006 |
| **Age** | -0.011 | 0.001 | 12.701 | 828.000 | 0.000 |
| **Gender: Women** | -0.016 | 0.034 | -0.470 | 828.000 | 0.638 |
| **Diagnosed: Yes** | 0.145 | 0.031 | 4.700 | 828.000 | 0.000 |
| **sd__(Intercept)** | 0.421 |  |  |  |  |
| **cor__(Intercept).Time** | -0.685 |  |  |  |  |
| **sd__Time** | 0.069 |  |  |  |  |
| **sd__Observation** | 0.486 |  |  |  |  |

**Interpersonal Sensitivity**

**
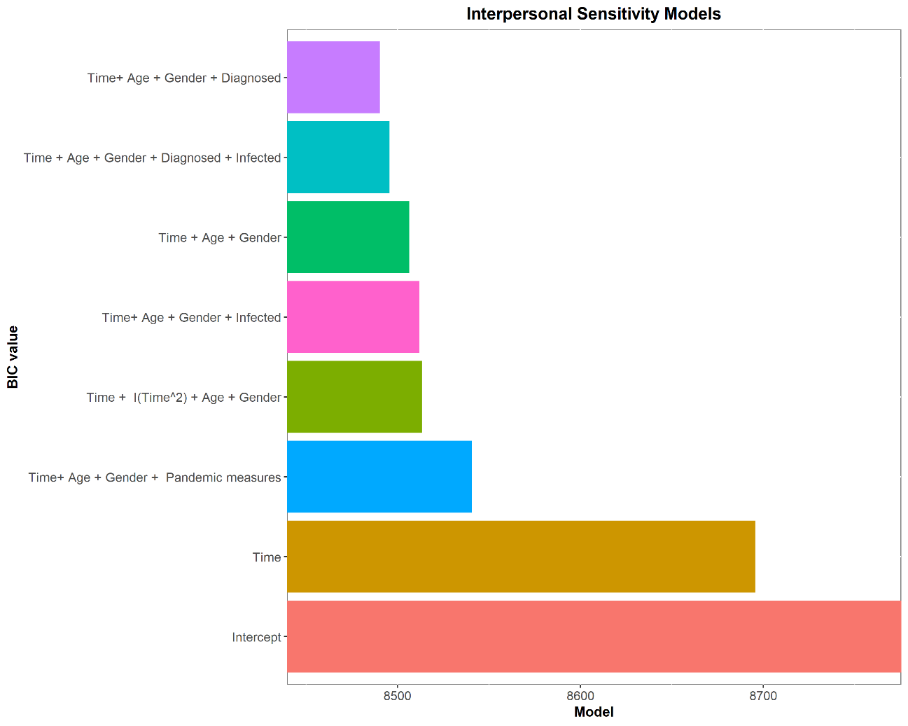
**

| **Linear mixed model: Interpersonal Sensitivity** | | | | | |
| --- | --- | --- | --- | --- | --- |
| **Term** | **Coefficient** | **SD** | **T value** | **df** | **p.** |
| **(Intercept)** | 1.184 | 0.074 | 16.046 | 1031.05 | 0.000 |
| **Time** | 0.051 | 0.007 | 6.858 | 831.00 | 0.000 |
| **Age** | -0.015 | 0.001 | 14.265 | 828.00 | 0.000 |
| **Gender: Women** | 0.111 | 0.040 | 2.744 | 828.00 | 0.006 |
| **Diagnosed: Yes** | 0.185 | 0.037 | 4.986 | 828.00 | 0.000 |
| **sd__(Intercept)** | 0.620 |  |  |  |  |
| **cor__(Intercept).Time** | -0.821 |  |  |  |  |
| **sd__Time** | 0.113 |  |  |  |  |
| **sd__Observation** | 0.573 |  |  |  |  |

**Obsession-Compulsion**

**
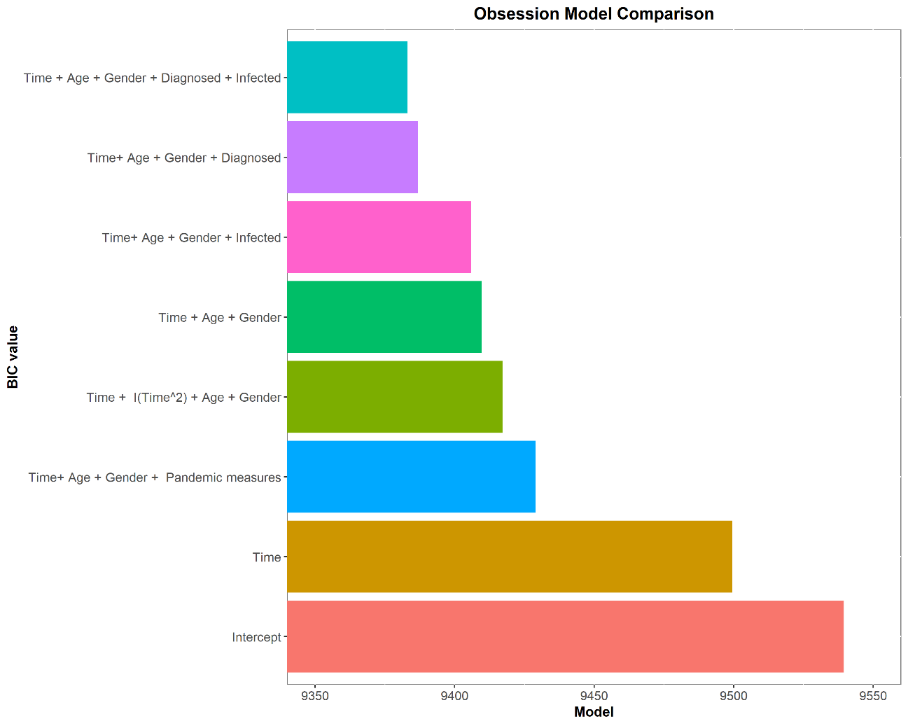
**

| **Linear mixed model: Obsession-Compulsion** | | | | | |
| --- | --- | --- | --- | --- | --- |
| **Term** | **Coefficient** | **SD** | **T value** | **df** | **p.** |
| **(Intercept)** | 1.368 | 0.086 | 15.921 | 1.015.905 | 0.000 |
| **Time** | 0.032 | 0.008 | 3.928 | 834.251 | 0.000 |
| **Age** | -0.011 | 0.001 | -9.456 | 827.423 | 0.000 |
| **Gender: Women** | 0.142 | 0.047 | 3.001 | 827.116 | 0.003 |
| **Diagnosed: Yes** | 0.247 | 0.043 | 5.675 | 827.072 | 0.000 |
| **infected** | -0.082 | 0.023 | -3.489 | 3.586.797 | 0.000 |
| **sd__(Intercept)** | 0.662 |  |  |  |  |
| **cor__(Intercept).Time** | -0.738 |  |  |  |  |
| **sd__Time** | 0.122 |  |  |  |  |
| **sd__Observation** | 0.630 |  |  |  |  |

**Paranoid Ideation**

**
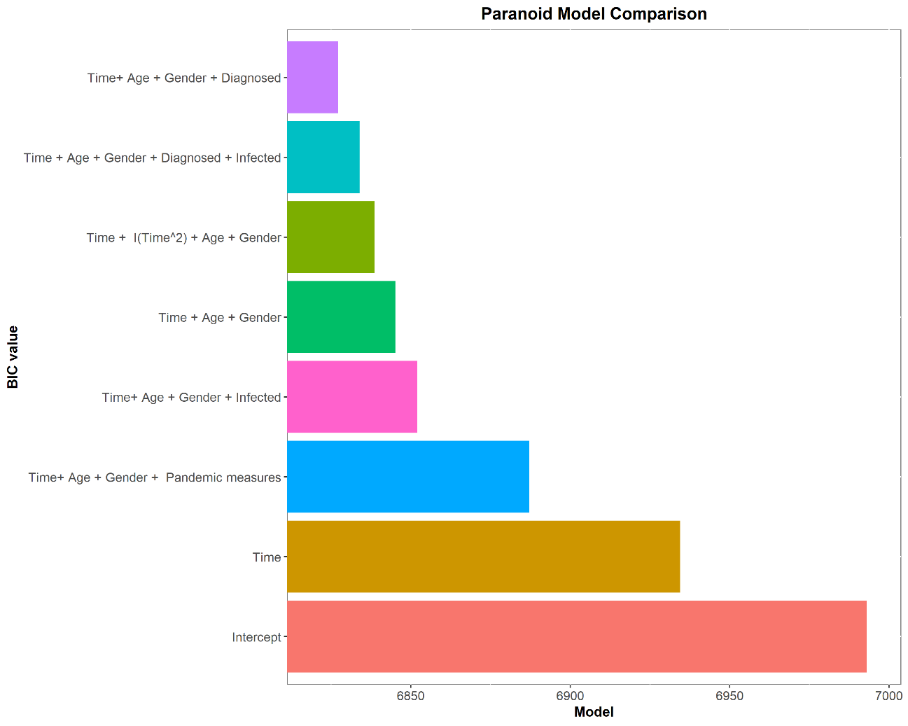
**

| **Linear mixed model: Paranoid Ideation** | | | | | |
| --- | --- | --- | --- | --- | --- |
| **Term** | **Coefficient** | **SD** | **T value** | **df** | **p.** |
| **(Intercept)** | 0.930 | 0.060 | 15.598 | 1.025.543 | 0.000 |
| **Time** | 0.032 | 0.006 | 5.406 | 831.001 | 0.000 |
| **Age** | -0.009 | 0.001 | -10.504 | 828.000 | 0.000 |
| **Gender: Women** | -0.017 | 0.033 | -0.520 | 828.000 | 0.603 |
| **Diagnosed: Yes** | 0.156 | 0.030 | 5.179 | 828.000 | 0.000 |
| **sd__(Intercept)** | 0.482 |  |  |  |  |
| **cor__(Intercept).Time** | -0.810 |  |  |  |  |
| **sd__Time** | 0.084 |  |  |  |  |
| **sd__Observation** | 0.474 |  |  |  |  |

**Phobic Anxiety**

**
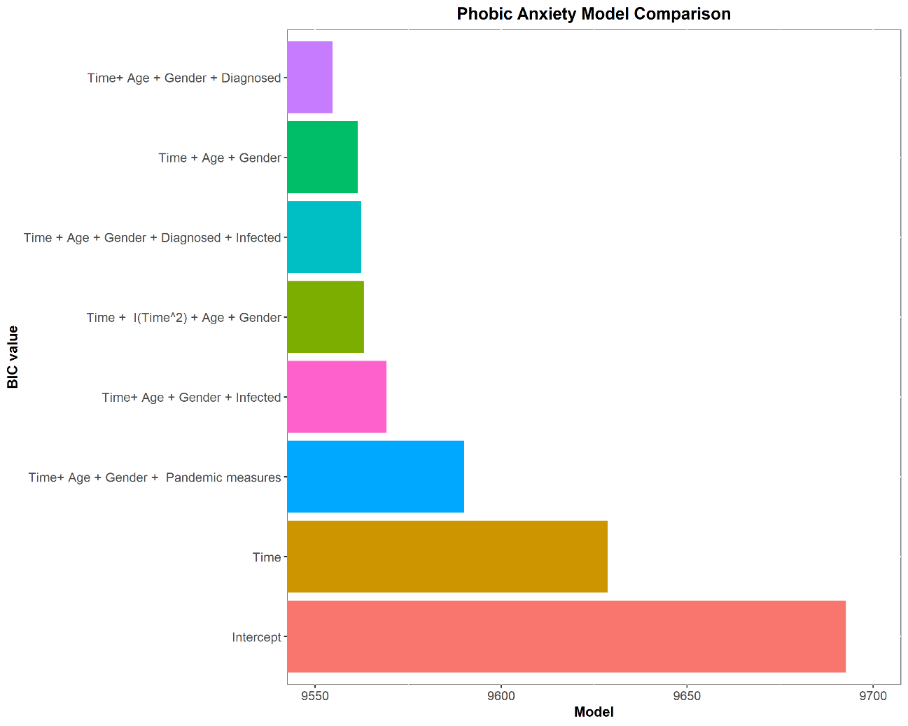
**

| **Linear mixed model: Phobic Anxiety** | | | | | |
| --- | --- | --- | --- | --- | --- |
| **Term** | **Coefficient** | **SD** | **T value** | **df** | **p.** |
| **(Intercept)** | 0.914 | 0.083 | 11.021 | 1.050.667 | 0.000 |
| **Time** | -0.014 | 0.009 | -1.606 | 831.000 | 0.109 |
| **Age** | -0.005 | 0.001 | -4.200 | 828.000 | 0.000 |
| **Gender: Women** | 0.330 | 0.045 | 7.291 | 828.000 | 0.000 |
| **Diagnosed: Yes** | 0.163 | 0.042 | 3.920 | 828.000 | 0.000 |
| **sd__(Intercept)** | 0.729 |  |  |  |  |
| **cor__(Intercept).Time** | -0.851 |  |  |  |  |
| **sd__Time** | 0.135 |  |  |  |  |
| **sd__Observation** | 0.652 |  |  |  |  |

**Psychoticism**

**
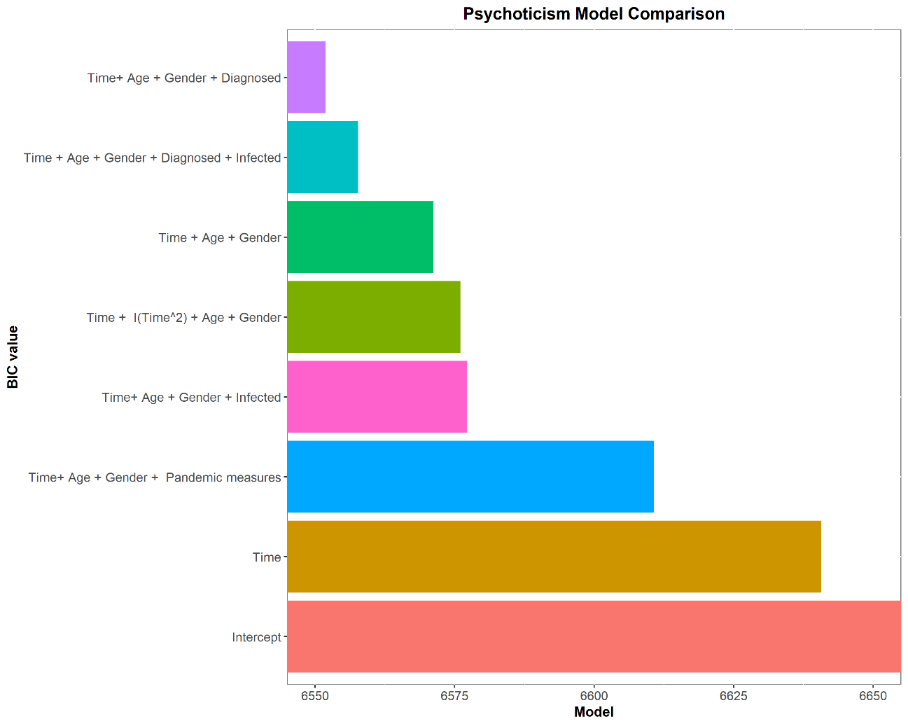
**

| **Linear mixed model: Psychoticism** | | | | | |
| --- | --- | --- | --- | --- | --- |
| **Term** | **Coefficient** | **SD** | **T value** | **df** | **p.** |
| **(Intercept)** | 0.893 | 0.059 | 15.106 | 1.010.944 | 0.000 |
| **Time** | -0.003 | 0.006 | -0.534 | 831.000 | 0.594 |
| **Age** | -0.008 | 0.001 | -9.143 | 828.000 | 0.000 |
| **Gender: Women** | 0.023 | 0.033 | 0.704 | 828.000 | 0.481 |
| **Diagnosed: Yes** | 0.160 | 0.030 | 5.333 | 828.000 | 0.000 |
| **sd__(Intercept)** | 0.470 |  |  |  |  |
| **cor__(Intercept).Time** | -0.794 |  |  |  |  |
| **sd__Time** | 0.078 |  |  |  |  |
| **sd__Observation** | 0.456 |  |  |  |  |

**Somatization**

**
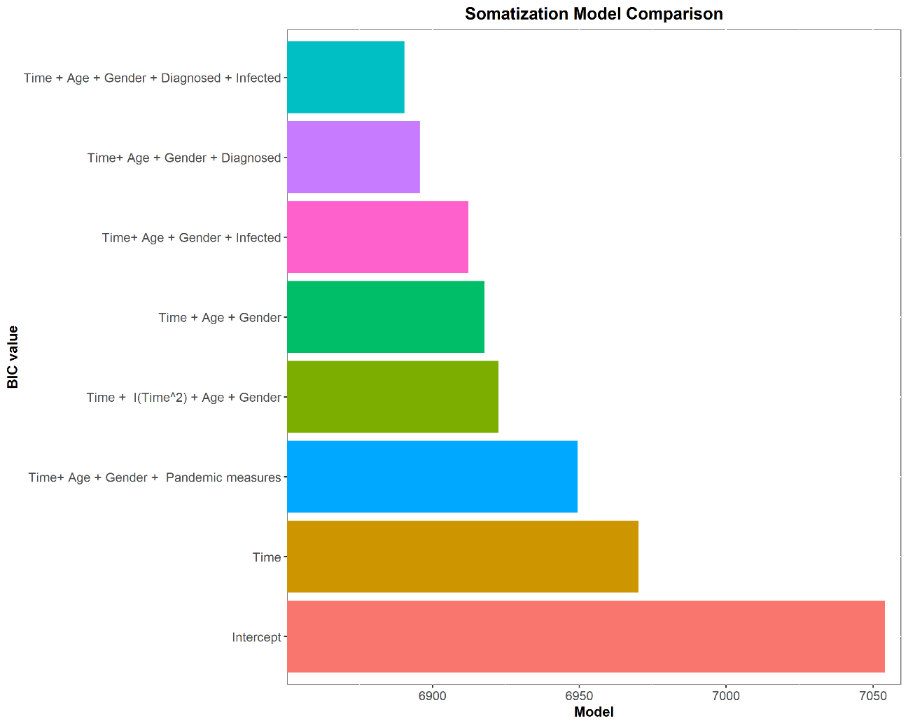
**

| **Linear mixed model: Somatization** | | | | | |
| --- | --- | --- | --- | --- | --- |
| **Term** | **Coefficient** | **SD** | **T value** | **df** | **p.** |
| **(Intercept)** | 0.487 | 0.063 | 7.710 | 1.116.912 | 0.000 |
| **Time** | 0.010 | 0.006 | 1.635 | 943.350 | 0.102 |
| **Age** | -0.004 | 0.001 | -4.084 | 827.946 | 0.000 |
| **Gender: Women** | 0.211 | 0.034 | 6.233 | 828.075 | 0.000 |
| **Diagnosed: Yes** | 0.173 | 0.031 | 5.555 | 827.773 | 0.000 |
| **infected** | 0.069 | 0.019 | 3.701 | 3.676.331 | 0.000 |
| **sd__(Intercept)** | 0.559 |  |  |  |  |
| **cor__(Intercept).Time** | -0.896 |  |  |  |  |
| **sd__Time** | 0.088 |  |  |  |  |
| **sd__Observation** | 0.473 |  |  |  |  |

**Figure S2.** Psychological Distress (GSI), the BSI-53 symptoms dimensions, COVID-19 related fear and Coping Skills during the pandemic over Time by Age range.

**
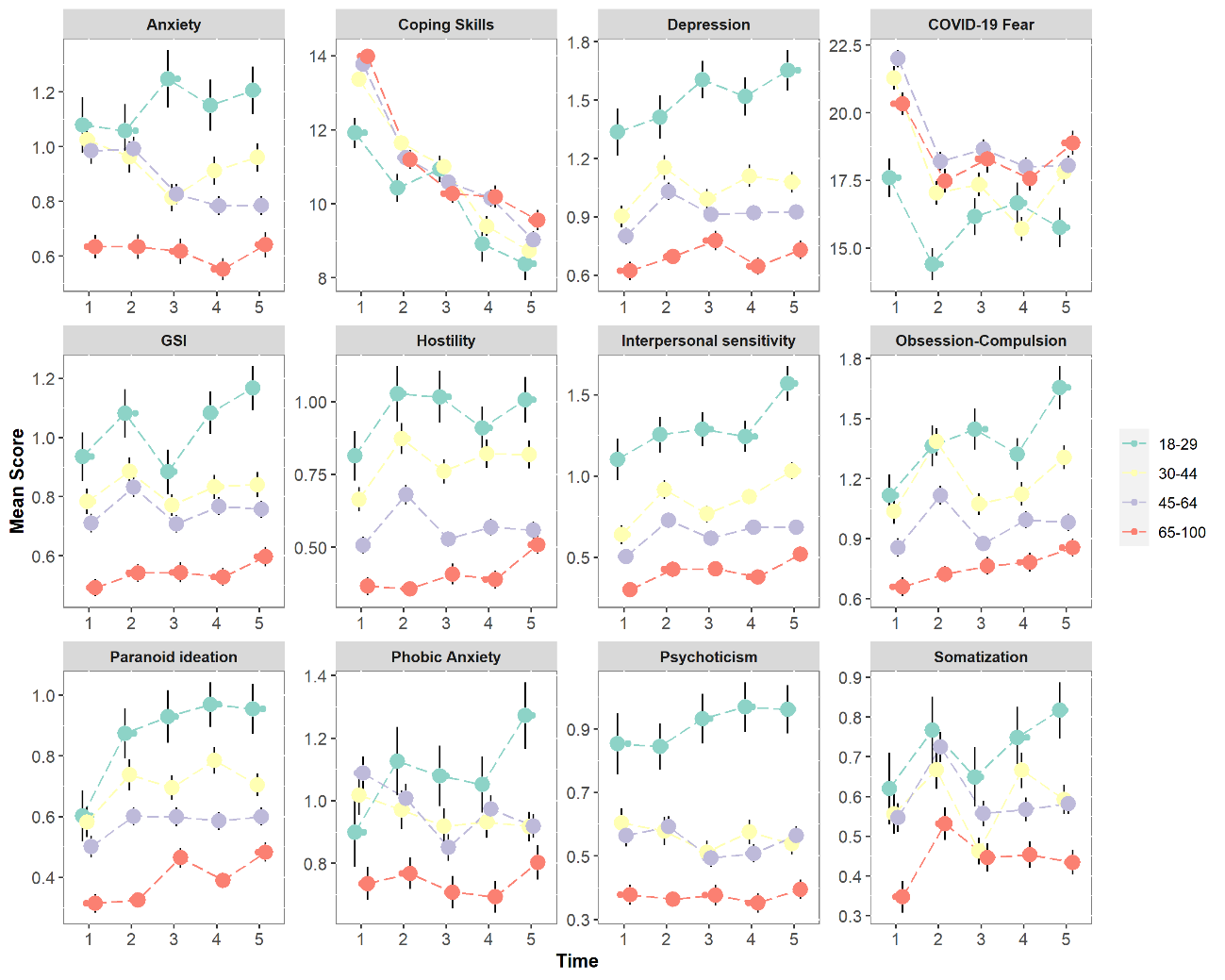
**

**Figure S3.** Psychological Distress (GSI), the BSI-53 symptoms dimensions, COVID-19 related fear and Coping Skills during the pandemic over Time by Gender.


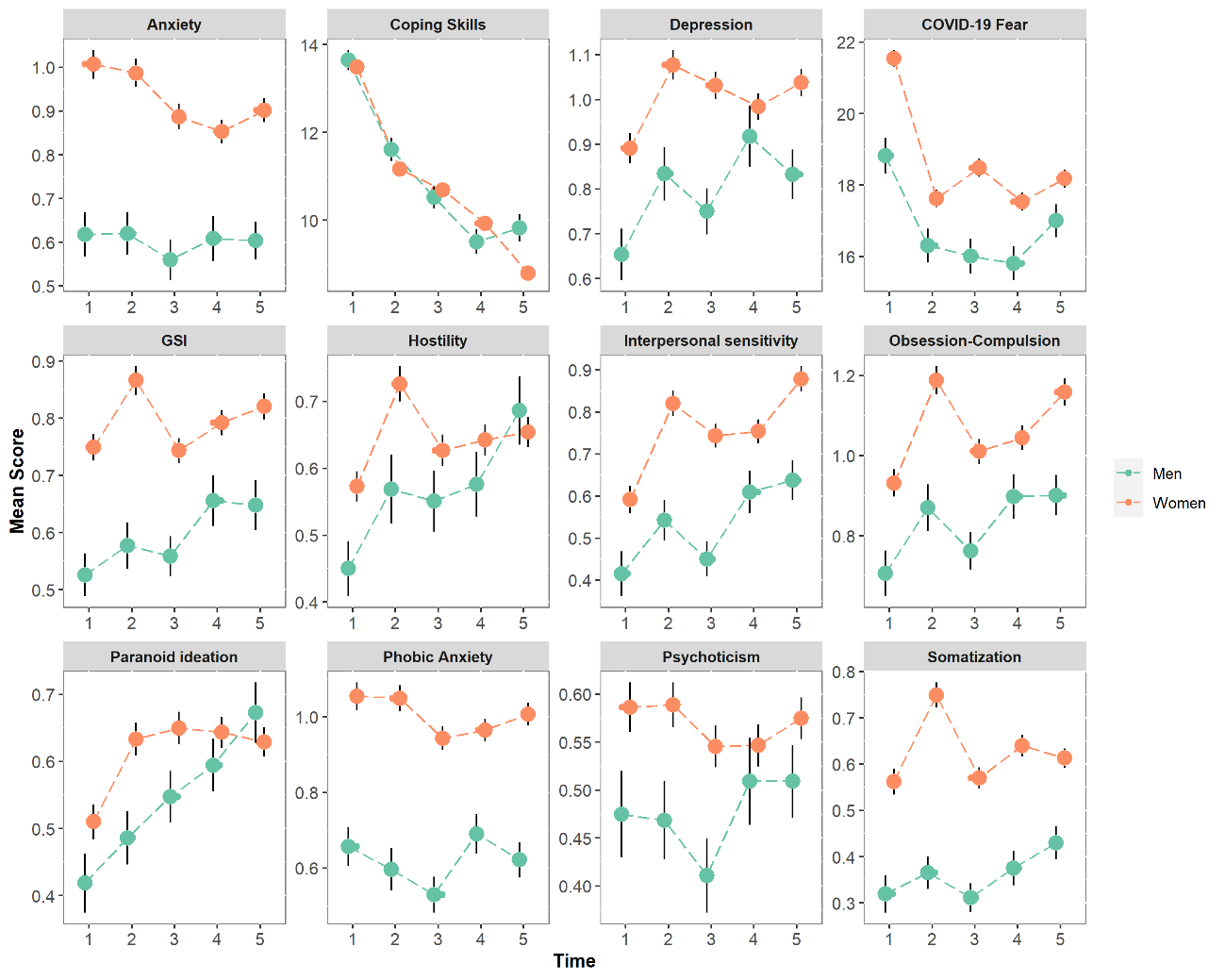


**Figure S4.** Psychological Distress (GSI), the BSI-53 symptoms dimensions, COVID-19 related fear and Coping Skills during the pandemic over Time by having a previous neuropsychiatric diagnosed.


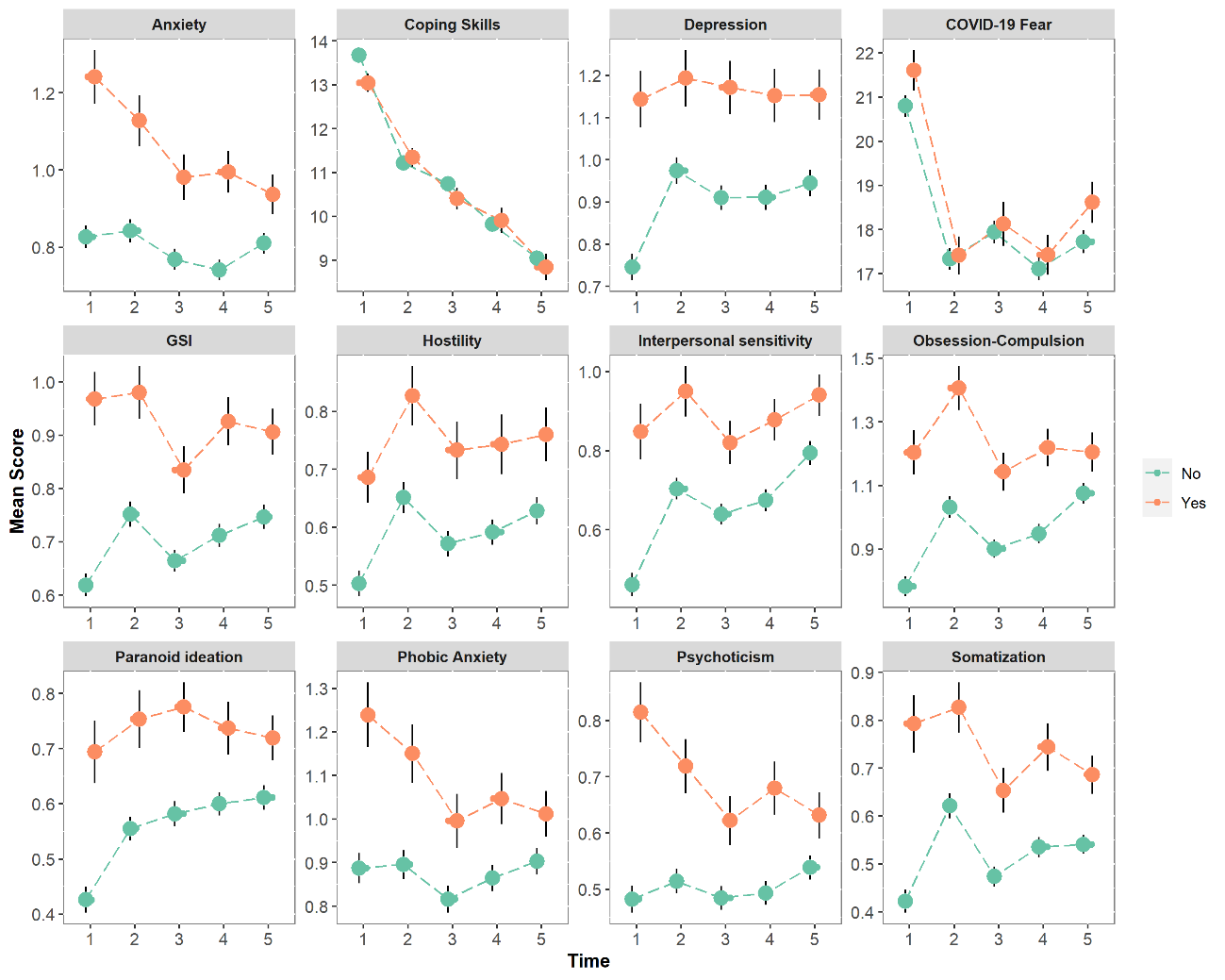


**Table S2.** Psychological Distress, symptom dimensions and changes in pandemic measures over Time (March 2020 – August 2021).

|  | **Time 1: April 2020** | **Time 2: July 2020** | **Time 3: October 2020** | **Time 4: March 2021** | **Time 5: August 2021** |
| --- | --- | --- | --- | --- | --- |
| **Covid-19 related Fear** | 20.996 (6.225) | 17.358 (6.233) | 17.988 (6.636) | 17.189 (6.428) | 17.944 (6.590) |
| **Coping Skills** | 13.524 (2.961) | 11.255 (3.303) | 10.659 (3.369) | 9.840 (4.177) | 9.002 (4.120) |
| **Somatization** | 0.513 (0.688) | 0.672 (0.687) | 0.518 (0.562) | 0.586 (0.584) | 0.576 (0.522) |
| **Obsession-Compulsion** | 0.886 (0.858) | 1.124 (0.890) | 0.961 (0.771) | 1.015 (0.783) | 1.107 (0.839) |
| **Interpersonal Sensitivity** | 0.556 (0.831) | 0.764 (0.760) | 0.684 (0.706) | 0.725 (0.712) | 0.830 (0.758) |
| **Depression** | 0.843 (0.836) | 1.028 (0.846) | 0.975 (0.772) | 0.971 (0.800) | 0.997 (0.794) |
| **Anxiety** | 0.928 (0.831) | 0.913 (0.802) | 0.821 (0.738) | 0.804 (0.697) | 0.842 (0.695) |
| **Hostility** | 0.548 (0.578) | 0.695 (0.683) | 0.612 (0.602) | 0.629 (0.604) | 0.661 (0.601) |
| **Phobic Anxiety** | 0.974 (0.933) | 0.958 (0.871) | 0.860 (0.802) | 0.909 (0.773) | 0.930 (0.756) |
| **Paranoid Ideation** | 0.492 (0.650) | 0.604 (0.606) | 0.629 (0.598) | 0.634 (0.578) | 0.638 (0.564) |
| **Psychoticism** | 0.564 (0.655) | 0.565 (0.589) | 0.518 (0.554) | 0.539 (0.570) | 0.562 (0.546) |
| **GSI** | 0.704 (0.599) | 0.808 (0.635) | 0.706 (0.548) | 0.765 (0.574) | 0.786 (0.582) |
| **Work change** |  |  |  |  |  |
| *- No* | - | 580 (69.7%) | 649 (78.0%) | 650 (78.1%) | 670 (80.5%) |
| *- Yes* | - | 252 (30.3%) | 183 (21.9%) | 182 (21.9%) | 162 (19.5%) |
| **Income reduction** |  |  |  |  |  |
| *- No* | - | 618 (74.3%) | 478 (57.5%) | 667 (80.2%) | 633 (76.1%) |
| *- Yes* | - | 214 (25.7%) | 325 (39.1%) | 165 (19.8%) | 199 (23.9%) |
| **Infected** |  |  |  |  |  |
| *- No* | 800 (96.2%) | 691 (83.1%) | 476 (57.2%) | 356 (42.8%) | 270 (32.5%) |
| *- Yes* | 32 (3.8%) | 141 (16.9%) | 356 (42.8%) | 476 (57.2%) | 562 (67.5%) |
| **Economic Concern** | 4.203 (0.921) | 4.273 (0.907) | 4.088 (1.130) | 4.240 (0.865) | 4.191 (0.897) |
| **Hygiene Measures** | 4.440 (0.712) | 4.320 (0.827) | 4.130 (1.157) | 4.228 (0.910) | 4.126 (0.967) |
| **Media Exposure** | 3.779 (0.878) | 3.553 (1.004) | 3.338 (1.055) | 3.185 (1.078) | 3.194 (1.077) |
| **Media Valuation** | 3.531 (0.839) | 3.347 (0.993) | 3.180 (1.070) | 3.111 (1.066) | 3.108 (1.066) |

*Mean value (SD) and frequency (percentage) when appropriate.

**Table S3.** Fit indices of Growth Mixture Model (GMM) comparison.

|  | **LogLik** | **Entropy** | **n Parameters** | **BIC** | **Class 1** | **Class 2** | **Class 3** | **Class 4** | **Class 5** | **Class 6** |
| --- | --- | --- | --- | --- | --- | --- | --- | --- | --- | --- |
| **Model 1** | -3239.203 | 1 | 1 | 6538.92 | 100 |  |  |  |  |  |
| **Model 2** | -3128.789 | 0.73 | 16 | 6365.159 | 18.02 | 81.97 |  |  |  |  |
| **Model 3** | -3031.018 | 0.85 | 23 | 6216.685 | 3.6 | 83.65 | 12.74 |  |  |  |
| **Model 4** | -2970.98 | 0.82 | 30 | 6140.675 | 73.9 | 10.81 | 6.78 | 8.53 |  |  |
| **Model 5** | -2937.373 | 0.83 | 37 | 6123.528 | 8.53 | 11.29 | 3 | 76.08 | 1.08 |  |
| **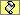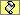Model 6** | -2907.883 | 0.8 | 44 | 6121.615 | 6.49 | 13.82 | 0.84 | 3 | 72.71 | 3.12 |

**Table S4.** Estimates for the GMM 4-class solution

|  | **Estimate** | **SE** | **Wald** | **p** |
| --- | --- | --- | --- | --- |
| **Intercept Class1** | 1.59 | 0.10 | 16.76 | 0.00 |
| **Intercept Class2** | 2.09 | 0.14 | 15.28 | 0.00 |
| **Intercept Class3** | 0.68 | 0.05 | 13.62 | 0.00 |
| **Intercept Class4** | 1.02 | 0.10 | 10.10 | 0.00 |
| **Time2 Class1** | 0.49 | 0.08 | 6.15 | 0.00 |
| **Time2 Class2** | -1.30 | 0.14 | -9.15 | 0.00 |
| **Time2 Class3** | 0.11 | 0.03 | 4.18 | 0.00 |
| **Time2 Class4** | 0.06 | 0.09 | 0.68 | 0.50 |
| **Time3 Class1** | -0.52 | 0.08 | -6.54 | 0.00 |
| **Time3 Class2** | 0.13 | 0.13 | 1.04 | 0.30 |
| **Time3 Class3** | 0.08 | 0.03 | 3.01 | 0.00 |
| **Time3 Class4** | 0.02 | 0.10 | 0.24 | 0.81 |
| **Time4 Class1** | -0.44 | 0.09 | -5.19 | 0.00 |
| **Time4 Class2** | -0.29 | 0.17 | -1.66 | 0.10 |
| **Time4 Class3** | 0.13 | 0.03 | 5.18 | 0.00 |
| **Time4 Class4** | 0.19 | 0.10 | 1.88 | 0.06 |
| **Time5 Class1** | -0.58 | 0.09 | -6.74 | 0.00 |
| **Time5 Class2** | -0.94 | 0.17 | -5.56 | 0.00 |
| **Time5 Class3** | 0.12 | 0.03 | 4.26 | 0.00 |
| **Time5 Class4** | 0.80 | 0.10 | 7.76 | 0.00 |
| **Age** | -0.01 | 0.00 | -7.48 | 0.00 |
| **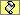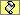Gender: Women** | 0.12 | 0.03 | 4.78 | 0.00 |

**Table S5.** Multinomial logistic regression results.

| **Class** | **Predictor** | **Odds Ratio** | **SD** | **Statistic** | **p** | **CI inf** | **CI sup** |
| --- | --- | --- | --- | --- | --- | --- | --- |
| Class 2: Fast Recovery | Age 45-64 | 0.22 | 0.31 | -4.96 | 0 | 0.12 | 0.4 |
|  | Age > 65 | 0.02 | 0.55 | -7.59 | 0 | 0.01 | 0.05 |
|  | Women | 2.28 | 0.24 | 3.43 | 0 | 1.42 | 3.65 |
|  | Essential Worker: Yes | 0.86 | 0.21 | -0.71 | 0.48 | 0.57 | 1.3 |
|  | Education: Low-Middle | 1.2 | 0.21 | 0.87 | 0.38 | 0.8 | 1.8 |
|  | Married | 0.52 | 0.22 | -2.94 | 0 | 0.34 | 0.8 |
|  | Unmarried-Widow/er | 0.55 | 0.25 | -2.41 | 0.02 | 0.34 | 0.9 |
|  | Diagnosed: Yes | 1.74 | 0.16 | 3.39 | 0 | 1.26 | 2.41 |
|  | Income: Upper | 0.86 | 0.25 | -0.62 | 0.53 | 0.53 | 1.39 |
|  | Income: Upper-Middle | 1.15 | 0.19 | 0.75 | 0.45 | 0.8 | 1.66 |
|  | Ocuppation: Others | 0.82 | 0.2 | -1.01 | 0.31 | 0.56 | 1.21 |
|  | Ocuppation: Student | 0.17 | 0.54 | -3.31 | 0 | 0.06 | 0.48 |
|  | Religious | 1.89 | 0.17 | 3.74 | 0 | 1.35 | 2.63 |
|  | Previous Trauma | 1.69 | 0.16 | 3.26 | 0 | 1.23 | 2.33 |
|  | Alcohol | 1.09 | 0.16 | 0.54 | 0.59 | 0.8 | 1.49 |
|  | Extroversion | 0.85 | 0.1 | -1.62 | 0.1 | 0.69 | 1.04 |
|  | Agreeableness | 0.91 | 0.12 | -0.82 | 0.41 | 0.72 | 1.14 |
|  | Conscientiousness | 1.16 | 0.11 | 1.38 | 0.17 | 0.94 | 1.43 |
|  | Neuroticism | 2.02 | 0.15 | 4.75 | 0 | 1.51 | 2.7 |
|  | Opennes | 0.97 | 0.1 | -0.35 | 0.72 | 0.8 | 1.17 |
|  | Resilience | 0.88 | 0.01 | -9.13 | 0 | 0.86 | 0.91 |
|  | Social Network Size | 0.95 | 0.01 | -5.42 | 0 | 0.94 | 0.97 |
| Class 3: Slow Recovery | Age 30-44 | 0.83 | 0.52 | -0.36 | 0.72 | 0.3 | 2.28 |
|  | Age 45-64 | 1.11 | 0.51 | 0.2 | 0.84 | 0.41 | 2.99 |
|  | Age > 65 | 0.62 | 0.57 | -0.85 | 0.39 | 0.2 | 1.88 |
|  | Women | 2.29 | 0.39 | 2.13 | 0.03 | 1.07 | 4.9 |
|  | Essential Worker: Yes | 0.47 | 0.41 | -1.85 | 0.06 | 0.21 | 1.04 |
|  | Education: Low-Middle | 0.87 | 0.3 | -0.45 | 0.65 | 0.48 | 1.57 |
|  | Married | 0.98 | 0.37 | -0.06 | 0.95 | 0.47 | 2.02 |
|  | Unmarried-Widow/er | 1.81 | 0.37 | 1.61 | 0.11 | 0.88 | 3.71 |
|  | Diagnosed: Yes | 2.59 | 0.25 | 3.74 | 0 | 1.57 | 4.27 |
|  | Income: Upper | 0.09 | 0.63 | -3.81 | 0 | 0.03 | 0.31 |
|  | Income: Upper-Middle | 0.44 | 0.36 | -2.28 | 0.02 | 0.21 | 0.89 |
|  | Ocuppation: Others | 1.1 | 0.32 | 0.31 | 0.76 | 0.59 | 2.06 |
|  | Ocuppation: Student | 6.63 | 0.5 | 3.8 | 0 | 2.5 | 6.5 |
|  | Religious | 1.46 | 0.26 | 1.46 | 0.14 | 0.88 | 2.41 |
|  | Previous Trauma | 2.88 | 0.24 | 4.35 | 0 | 1.79 | 4.64 |
|  | Alcohol | 0.72 | 0.25 | -1.3 | 0.19 | 0.44 | 1.18 |
|  | Extroversion | 0.73 | 0.17 | -1.89 | 0.06 | 0.53 | 1.01 |
|  | Agreeableness | 0.46 | 0.18 | -4.27 | 0 | 0.32 | 0.66 |
|  | Conscientiousness | 1.61 | 0.15 | 3.29 | 0 | 1.21 | 2.14 |
|  | Neuroticism | 4.27 | 0.23 | 6.34 | 0 | 2.73 | 6.69 |
|  | Opennes | 0.92 | 0.15 | -0.61 | 0.54 | 0.69 | 1.22 |
|  | Resilience | 0.9 | 0.02 | -5.54 | 0 | 0.87 | 0.93 |
|  | Social Network Size | 0.95 | 0.01 | -3.46 | 0 | 0.93 | 0.98 |
| Class 4: Deteriorating | Age 30-44 | 0.25 | 0.33 | -4.16 | 0 | 0.13 | 0.48 |
|  | Age 45-64 | 0.32 | 0.32 | -3.56 | 0 | 0.17 | 0.6 |
|  | Age > 65 | 0.08 | 0.39 | -6.51 | 0 | 0.04 | 0.17 |
|  | Women | 1.95 | 0.22 | 3.01 | 0 | 1.26 | 3 |
|  | Essential Worker: Yes | 0.78 | 0.23 | -1.12 | 0.26 | 0.5 | 1.21 |
|  | Education: Low-Middle | 1.6 | 0.19 | 2.42 | 0.02 | 1.09 | 2.33 |
|  | Married | 1.14 | 0.27 | 0.49 | 0.62 | 0.67 | 1.95 |
|  | Unmarried-Widow/er | 1.07 | 0.3 | 0.22 | 0.83 | 0.59 | 1.93 |
|  | Diagnosed: Yes | 0.75 | 0.19 | -1.45 | 0.15 | 0.51 | 1.1 |
|  | Income: Upper | 1.89 | 0.21 | 3.02 | 0 | 1.25 | 2.87 |
|  | Income: Upper-Middle | 1.04 | 0.19 | 0.21 | 0.83 | 0.71 | 1.52 |
|  | Ocuppation: Others | 1.14 | 0.2 | 0.68 | 0.5 | 0.78 | 1.68 |
|  | Ocuppation: Student | 1.06 | 0.36 | 0.16 | 0.87 | 0.52 | 2.14 |
|  | Religious | 2.03 | 0.18 | 3.94 | 0 | 1.43 | 2.89 |
|  | Previous Trauma | 0.65 | 0.19 | -2.21 | 0.03 | 0.44 | 0.95 |
|  | Alcohol | 1.19 | 0.16 | 1.12 | 0.26 | 0.87 | 1.63 |
|  | Extroversion | 1.03 | 0.1 | 0.29 | 0.77 | 0.84 | 1.26 |
|  | Agreeableness | 1.58 | 0.12 | 3.86 | 0 | 1.25 | 1.99 |
|  | Conscientiousness | 1.36 | 0.1 | 3.09 | 0 | 1.12 | 1.65 |
|  | Neuroticism | 1.81 | 0.14 | 4.11 | 0 | 1.36 | 2.4 |
|  | Opennes | 1.34 | 0.09 | 3.19 | 0 | 1.12 | 1.6 |
|  | Resilience | 0.97 | 0.01 | -2.28 | 0.02 | 0.94 | 1 |
|  | Social Network Size | 0.96 | 0.01 | -4.65 | 0 | 0.94 | 0.98 |
|  | Agreeableness | 1.62 | 0.13 | 3.83 | 0 | 1.27 | 2.07 |
|  | Conscientiousness | 1.3 | 0.1 | 2.59 | 0.01 | 1.07 | 1.6 |
|  | Neuroticism | 2 | 0.15 | 4.62 | 0 | 1.49 | 2.68 |
|  | Opennes | 1.32 | 0.09 | 2.97 | 0 | 1.1 | 1.59 |
|  | Resilience | 0.97 | 0.01 | -2.39 | 0.02 | 0.94 | 0.99 |
| 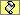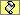 | Social Network Size | 0.94 | 0.01 | -5.96 | 0 | 0.93 | 0.96 |

*Class 1 “Resilient”, served as reference.

**Figure S5.** Changes in pandemic measures over time according to class membership.


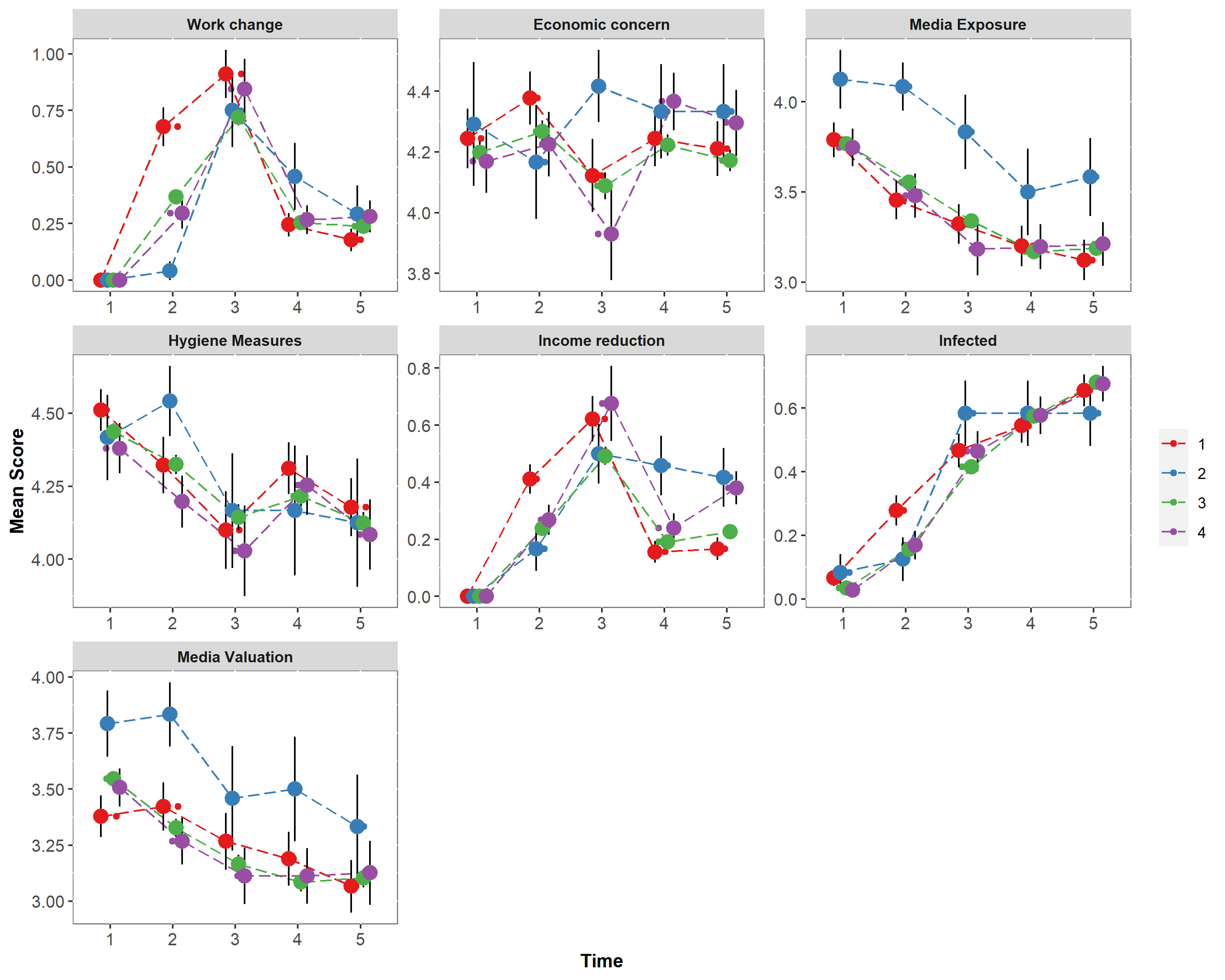

Supplement: Supplementary file 1 — Supplementary Information. [file 41598_2022_9663_MOESM1_ESM.docx]
